# Parenteral prostacyclin utilization in patients with pulmonary arterial hypertension in the intermediate-risk strata: a retrospective chart review and cross-sectional survey

**DOI:** 10.1186/s12890-024-03388-w

**Published:** 2024-11-20

**Authors:** Anjali Vaidya, Margaret R. Sketch, Meredith Broderick, Oksana A. Shlobin

**Affiliations:** 1 Temple University Lewis Katz School of Medicine, Philadelphia, PA USA; 2 United Therapeutics Corporation, Research Triangle Park, NC USA; 3 Inova Fairfax Hospital, University of Virginia School of Medicine, 3300 Gallows Road, IHVI, Falls Church, VA 22042 USA

**Keywords:** Retrospective studies, Prostacyclins, Prostaglandins, Risk assessment, Pulmonary hypertension

## Abstract

**Background:**

Current clinical guidelines support use of parenteral prostacyclin therapy for patients with pulmonary arterial hypertension (PAH) at intermediate risk. The objective of this study was to assess parenteral prostacyclin therapy use among patients at intermediate risk according to the Comparative, Prospective Registry of Newly Initiated Therapies for Pulmonary Hypertension (COMPERA) 2.0 four-strata risk assessment model.

**Methods:**

This was a retrospective chart review and cross-sectional online survey of healthcare professionals (HCPs). Included patients were classified as intermediate-low or intermediate-high risk per COMPERA 2.0 between 2016 and 2020 (index visit), initiated on a parenteral prostacyclin any time following intermediate risk assessment, and had World Health Organization (WHO) Functional Class (FC), 6-minute walk distance (6MWD), and B-type natriuretic peptide/N-terminal pro B-type natriuretic peptide (BNP/NT-proBNP) assessments at index and first comprehensive follow-up visits (follow-up).

**Results:**

A total of 139 HCPs (53% community-based, 47% Pulmonary Hypertension Care Center-based) participated in the survey and provided 350 patient records; among these, mean age (SD) was 54.1 (15.3) years and 52% were female. Median (IQR) time from parenteral prostacyclin initiation to follow-up was 3.0 months (2.0, 7.0). At parenteral prostacyclin initiation for the 280 patient records with available COMPERA 2.0 assessments, 62% of patients were intermediate-high risk, 33% were intermediate-low risk and 3% were low risk, improving to 38%, 53%, and 8%, respectively, at follow-up.

**Conclusions:**

Improvements were seen for the individual COMPERA 2.0 risk calculator parameters and for several other clinical parameters. Findings from this study substantiate recent guidelines suggesting earlier use of this treatment in intermediate-risk patients with PAH.

**Clinical trial number:**

Not applicable.

**Supplementary Information:**

The online version contains supplementary material available at 10.1186/s12890-024-03388-w.

## Background

Pulmonary arterial hypertension (PAH) is a serious disease associated with a broad range of medical conditions involving the pulmonary circulation [1]. PAH is progressive, leading to right heart failure and is typically fatal [2, 3]. Managing PAH can be complex, as risk assessments to predict patient outcomes include various clinical, laboratory, right heart imaging and catheterization hemodynamic parameters as well as exercise and functional status [2, 4]. Risk stratification tools can aid healthcare professionals in managing and treating their patients with PAH by formally evaluating their risk of death in one year, but the number of available tools and lack of consensus on the best approach adds to the complexity of PAH management. Risk assessment tools for PAH have evolved over the past decade and now also include the Registry to Evaluate Early and Long-Term PAH Disease Management (REVEAL) [5], REVEAL 2.0 [6], and REVEAL Lite 2 [7]. As with previous risk stratification methods, the 2022 European Society of Cardiology (ESC) and the European Respiratory Society (ERS) guidelines [2] include a three-stratum baseline risk score calculator, derived from the Swedish PAH Registry [8, 9]. Because intermediate clinical risk represents a majority of patients (approximately 70%) [9], and many patients in the European registries remained in the intermediate-risk stratum at follow-up, a method differentiating the intermediate-risk category into intermediate-low and intermediate-high risk was developed to guide effective treatment approaches and understand delineation within the broader risk category [10]. The Comparative, Prospective Registry of Newly Initiated Therapies for Pulmonary Hypertension (COMPERA) 2.0 is a four-stratum risk assessment model using the three non-invasive parameters identified from the French PH Registry [9–11]. 

Parenteral prostacyclin therapy is an effective treatment option for PAH [12–17] but many patients never receive this therapy [18–22]. Until recently, clinical guidelines only recommended treatment with prostacyclin analogue therapy as initial treatment for patients with more severe and rapidly progressing PAH [23, 24]. However, recent guidelines from ESC/ERS in 2022 support use of parenteral prostacyclin therapy as an adjunct therapy for patients with PAH who present at high risk status or stay at intermediate-high risk status after an initial therapy is instituted [2]. In an editorial discussing upfront combination therapy for PAH, Cascino and McLaughlin suggest that parenteral prostacyclin therapy be considered as initial therapy for appropriate patients at intermediate risk, as well as at three or six-month follow-up [25]. 

The primary goal of this research was to determine the use of parenteral prostacyclin therapy among patients with PAH at intermediate risk of death in one year according to the COMPERA 2.0 four-strata risk assessment model. Specifically, we sought to (1) understand the impact of timing of escalation to parenteral prostacyclin therapy on clinical outcomes in an intermediate risk patient population, (2) differentiate and understand utilization and clinical outcomes in patients who receive parenteral prostacyclin therapy as intermediate-low or intermediate-high risk, (3) assess healthcare professionals’ (HCP) decisions regarding escalation of therapy to include a parenteral prostacyclin, (4) quantify HCPs’ perceptions regarding utilization of parenteral prostacyclin for intermediate-risk patients, and (5) understand HCPs’ attitudes and treatment decisions regarding PAH management.

## Methods

### Study design

This study consisted of a cross-sectional survey and a retrospective chart review, conducted in the United States between September 27, 2022, and March 30, 2023. HCPs completed an online survey and provided anonymized patient chart data for their patients with PAH. The study instruments were developed by the authors and a third-party vendor (KJT Group, Inc., Rochester, NY, USA). The survey was conducted by KJT Group, Inc. The survey and chart review form were pretested among six HCPs (three pulmonologists, two cardiologists, and one nurse practitioner with a specialty in cardiology) using web-assisted telephone interviews to assess clarity and face validity. Minor revisions were made to the survey and chart review form based on feedback from the pretests.

Study participants were recruited via email or postal mail across the United States. Potential respondents contacted via email were recruited through online panel companies (SampleNinja LLC and Dynata LLC) with which they provided permission to be contacted for research purposes. Additionally, a postal mailing with a letter from KJT Group, Inc. was sent to a list of HCPs from 74 accredited PH Care Centers. Follow-up reminders were sent to non-responders via postal mail, email, and/or telephone to maximize the response rate. The study invitations described the nature of the study and included a link to Decipher, a secure online survey platform, to self-administer the survey and complete the chart reviews. Respondents who electronically consented to participate in the study were allowed to enter the screening portion of the survey. Eligible participants completing the entire survey received a modest monetary incentive; they also received an incentive for completing the chart forms. Sample sizes were determined based on estimated feasibility and prior research with HCPs who treat patients with PAH.

The study protocol was submitted to WCG Institutional Review Board for ethical review and was determined to be exempt because the research included survey procedures with adequate provisions to protect the privacy of subjects and maintain data confidentiality.

### Healthcare professional selection criteria

HCPs were included in the study if they met the following criteria: were a physician, nurse practitioner, physician assistant, or advanced practice registered nurse; specialized in pulmonology, cardiology, or rheumatology, practiced in the United States but not Vermont (to comply with Sunshine Act reporting requirements); in practice for at least two years; currently treating at least 20 patients with PAH (Group 1 PH); initiated treatment for at least ten patients with PAH in the past 12 months; currently treating at least five patients with PAH at intermediate risk; and personally initiated or oversaw the initiation of parenteral prostacyclin therapy.

### Healthcare professional survey

The 20-minute survey (Supplementary Material 1) consisted of questions regarding HCPs’ patient population; PAH diagnosis, risk assessment, monitoring practices; and attitude and approach to PAH treatment. The questions included open text (unaided response), numeric entry (proportion of patients) multiple-choice, five-point Likert-scales from one (very unlikely/no influence at all/strongly disagree) to five (very likely/great deal of influence/strongly agree), and 11-point Likert-scales from 0 (not at all important/do not at all meet my needs) to ten (extremely important/completely meets my needs). A maximum difference scaling exercise was also included in the survey. This exercise consisted of 11 attributes (including factors related to survival, clinical efficacy, risk status, safety profile, compliance, insurance coverage, and dosing flexibility) that might influence HCPs’ decision making when prescribing a therapy for the treatment of PAH to their intermediate-risk patients. A total of ten choice tasks were presented to each participant in which they were asked to choose the most and least important attribute.

### Patient chart review

Participating HCPs were asked to complete chart review forms for a minimum of two and up to ten patients with Group 1 PH. Patients were included in the chart review if they (1) were categorized as intermediate-low (score = 2) or intermediate-high risk (score = 3) per COMPERA 2.0 risk stratification model between 2016 and 2020 (index visit); (2) were initiated on a parenteral prostacyclin any time following the index visit; (3) did not receive an experimental therapy at any point after the index visit; (4) received a comprehensive follow-up visit (follow-up) after parenteral prostacyclin was initiated; and (5) had the following assessments at index and follow-up: World Health Organization (WHO)/New York Heart Association (NYHA) Functional Class (FC), 6-minute walk distance (6MWD), and B-type natriuretic peptide/N-terminal pro B-type natriuretic peptide (BNP/NT-proBNP).

After an initial sample patient charts, an oversample of patient charts were collected to obtain additional data, which included the requirement of a follow-up visit date and COMPERA 2.0 parameters at parenteral prostacyclin initiation. Additionally, the time period during which the follow-up visit occurred was modified to be 6–12 months following parenteral prostacyclin initiation.

Patient demographic information was collected at the time of patients’ intermediate risk classification. Clinical characteristics were captured at the time of patients’ intermediate risk classification and at the follow-up visit. Details of patients’ parenteral prostacyclin therapy history following their intermediate risk classification were captured, including treatment type, timing, and reasons for initiation. PAH treatment history from diagnosis prior to index date to follow-up was also collected. Risk status per COMPERA 2.0 from parenteral prostacyclin therapy initiation to follow-up was assessed. Participating HCPs entered clinical values directly into the chart forms or were asked to select from categorial response options. The only information calculated from the inputted data was intermediate risk status per COMPERA 2.0.

### Qualitative interviews

Following the initial quantitative survey, 45-minute qualitative interviews were conducted between October 23, 2023 and November 3, 2023. Respondents were recruited from the HCPs who completed the survey and patient chart portion of the study. At the end of the survey, HCPs were asked about their interest in participating in interviews at a later date. The purpose of the qualitative portion of the study was to obtain additional context and insights about the survey and chart review findings.

### Statistical analyses

We performed descriptive statistical analyses (means, frequencies) of the aggregated data using Q Research Software for Windows (A Division of Displayr, Inc., New South Wales, Australia). Categorical data are expressed as frequencies and proportions. Continuous and count variables are presented using mean, standard deviation (SD), median, and interquartile range (IQR). Missing data and outliers were removed from calculations and were not imputed. Responses to open-ended questions were coded, categorized thematically, and quantified. PAH risk status was calculated using COMPERA 2.0, REVEAL 2.0, and REVEAL Lite 2 for the patient records for which the risk calculator parameters were available. Qualitative data was analyzed thematically; the analysis consisted of coding the transcripts to identify emerging themes and constructs.

Dosing data was collected as an exploratory endpoint for the chart review. However, upon review of the data, it was determined that there were a substantial number of “outliers” that were largely inconsistent with clinical plausibility, which may have been due to inconsistent reporting/documentation. Thus, the dosing data is not reported in this analysis, but information from the qualitative interviews which sought to understand how PAH treatments are typically dosed and documented in the electronic health record (EHR) is included.

Tests of differences (*t*-tests for means and *z*-tests for proportions) for subgroup analyses by practice setting (community-based and accredited PH centers) were performed using Q Research Software tables for the HCP survey results. Statistical significance was set at *p* < 0.05, using 2-tailed tests. Hierarchical Bayes modeling was used to generate the mean relative importance score for each attribute assessed in the maximum difference scaling exercise in the HCP survey. Attribute scores are positive values ranging from 0 to 100, that sum to 100. The scores have the valuable property of ratio-scaling, meaning for example, that an attribute with a rating of 20 is twice as important as an attribute with a rating of 10.

## Results

### Healthcare professional sample

A total of 1,289 HCP respondents entered the survey, of which 139 qualified for, and participated in this study. The remaining respondents did not complete the survey, did not qualify, or were over the target quota. The sample was comprised primarily of HCPs specializing in cardiology or pulmonology and was similarly split between those affiliated with PH centers and those who were community-based. Sample characteristics of healthcare providers are shown in Table 1.

**Table 1:** Characteristics of healthcare professional participants

Characteristics	Healthcare professionals (*N* = 139)
Age, mean ± SD	49.4 ± 8.4
Years in practice, mean ± SD	16.2 ± 6.3
Role, n (%)	
Physician	134 (96%)
NP/PA/APRN	5 (4%)
Specialty, n (%)	
Cardiology	72 (52%)
Pulmonology	50 (36%)
Rheumatology	17 (12%)
Affiliation, n (%)	
Community-based	73 (53%)
PH center	66 (47%)
Hospital affiliation type, n (%)	
Major medical center, university/research hospital, or tertiary referral center	57 (41%)
Community teaching hospital	39 (28%)
Community non-teaching hospital	39 (28%)
None of the above / practice is not affiliated with a hospital	4 (3%)
Region, n (%)	
Northeast	34 (24%)
Midwest	26 (19%)
South	44 (32%)
West	35 (25%)
Practice setting	
Urban	76 (55%)
Suburban	59 (42%)
Rural	4 (3%)
Number of PAH patients personally initiated treatment for in past year, median (IQR)	50 (30.0, 90.0)

Abbreviations: APRN, advanced practice registered nurse; NP, nurse practitioner; PA, physician assistant; PAH, pulmonary arterial hypertension; PH, accredited pulmonary hypertension center; SD, standard deviation; IQR, interquartile range

The qualitative phase of this research included 15 HCPs agreeing to participate in the 45-minute telephone interviews. All of the HCPs participating in the qualitative interviews were physicians; five were cardiologists; five were pulmonologists, and five were rheumatologists. Affiliation was split between community-based practices (*n* = 8) and PH centers (*n* = 7). Additionally, representation was similar across regions (*n* = 3, Northeast; *n* = 5 Midwest; *n* = 3 South; *n* = 4 West).

### Healthcare professional survey

#### PAH patient population

The survey asked HCPs about their overall PAH patient population. HCPs surveyed reported that of their patients with PAH (Group 1 PH) whom they currently manage, 29% would be classified as low risk, 42% as intermediate risk, and 29% as high risk based on their clinical judgment; HCP-reported risk stratification was similar among those practicing in PH centers and those who were community-based. HCPs indicated that of their intermediate-risk patients whom they currently manage, they would classify most as unchanged (36%) or improving (38%), with one-quarter (25%) declining. When asked about the WHO/NYHA FC of their intermediate-risk patients, HCPs reported that most were FC II or III at diagnosis (28% and 32%, respectively); 21% were FC I, 17% were FC IV, and 2% were unknown.

Regarding their overall PAH patient population, HCPs reported that of their patients being treated with a prostacyclin (any route of administration), 22% were low risk, 41% were intermediate-risk, and 37% were high risk prior to starting prostacyclin therapy. For patients on a parenteral prostacyclin, 18% were low risk, 35% were intermediate risk, and 47% were high risk prior to starting this therapy. As reported by HCPs, on average, 56% of their patients were initiated on non-parenteral prostacyclin therapy prior to escalating to a parenteral prostacyclin, with an average of 13 months between initiation of the two treatment types. HCPs reported that of their patients at intermediate risk, 27% were currently being treated with monotherapy, 40% with a dual combination therapy, and 27% with at least three PAH-specific drugs; 6% were not treated with any PAH-specific therapy.

#### PAH risk assessment and monitoring

On average, the HCPs surveyed reported performing a formal risk assessment via a risk stratification method in 58% of their PAH patient visits. Among those conducting formal risk assessments, HCPs reported using ESC/ERS guidelines 37% of the time; REVEAL 2.0 and REVEAL Lite 2 were used less frequently (17% and 12% of the time, respectively). COMPERA 2.0 was also infrequently used (6% of the time among all HCPs) but use was significantly higher among HCPs practicing in PH centers than those in community-based practices (10% vs. 3%, *p* < 0.05). HCPs reported using “gestalt/clinical impression” 19% of the time, driven primarily by those in community-based practices (25% vs. 11% among those in PH centers, *p* < 0.05). The main reasons cited (unaided) by HCPs for not conducting formal risk assessments at every visit with their PAH patients include patient stability (20%), assessments conducted informally (17%), and formal assessments being time-consuming (15%).

When inquiring about awareness of clinical guidelines, only three of the 15 HCPs interviewed qualitatively reported being aware of the changes to the 2022 ESC/ERS guidelines recommending that parenteral prostacyclin therapy be considered in patients who present at intermediate-high or high risk. However, some HCPs (*n* = 6) noted the new recommendations would not change their prescribing patterns because it is in line with their current practice; others stated that patient willingness and symptom assessment will continue to drive their treatment decisions.

HCPs surveyed reported monitoring their patients’ response to PAH-specific drug therapy using various methods. Vital signs were used most frequently, with nearly all (93%) reporting using this method at least every six months, followed by WHO/NYHA FC (85%), 6MWD (71%) and BNP/NT-proBNP (70%). Although most HCPs (80%) reported using echocardiograms every six or 12 months, differences were seen in the frequent use of echocardiograms (every three months) between HCPs practicing in PH centers (17%) and community-based practices (3%).

During the qualitative interviews, participants were asked how long they typically wait to assess the treatment progress of their patients not on a parenteral prostacyclin before escalating treatment. The period varied between every six weeks to six months, with several participants (*n* = 7) indicating the assessments occur between two to three months on current therapy. However, some HCPs (*n* = 6) also mentioned that they re-evaluate patients sooner in case of active worsening.

#### Attitudes and approaches towards PAH treatment

**Fig. 1:**
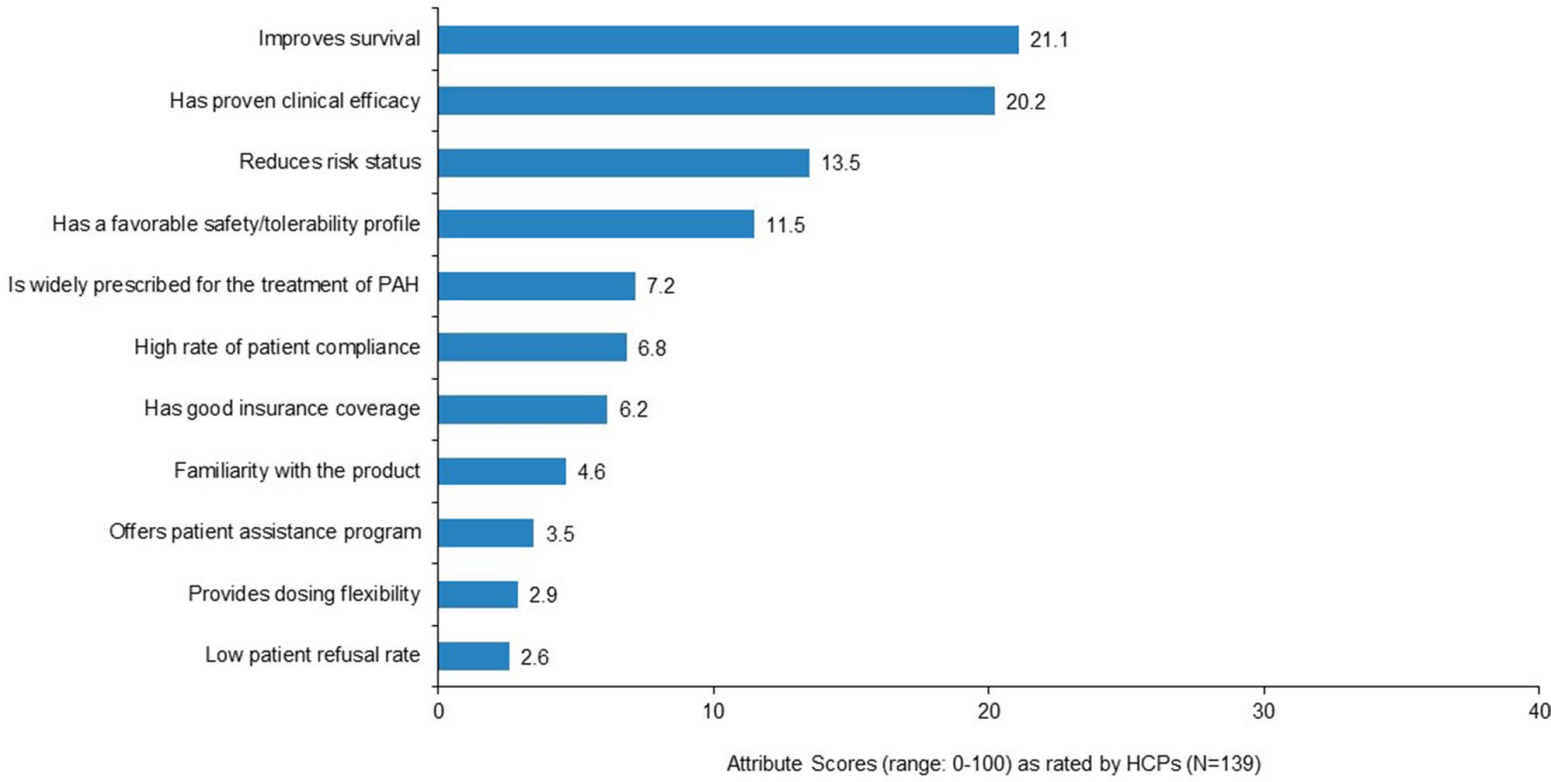
Importance of attributes influencing healthcare professionals’ PAH treatment decisions for patients at intermediate risk. Note: Responses to the survey question “We would now like you to consider several attributes that might influence your decision when prescribing a therapy for the treatment of PAH to your intermediate-risk patients. Considering only the attributes listed on the screen, please select the attribute that is most important in your therapy selection and then select the least important attribute.” Maximum difference scaling output consists of a rank ordering of attributes along with standard scores that represent the strength (magnitude) of the associated importance. Attribute scores are positive values ranging from 0 to 100, that sum to 100. The scores have the valuable property of ratio-scaling, meaning for example, that an attribute with a rating of 20 is twice as important as an attribute with a rating of 10. Abbreviations: HCP, healthcare provider; PAH, pulmonary arterial hypertension

As assessed by the maximum difference scaling exercise, the most important attributes to HCPs when prescribing a therapy to patients with PAH at intermediate risk were “improves survival” and “has proven clinical efficacy.” Low patient refusal rate and dosing flexibility were least important **(**Fig. 1**)**.

When asked about the extent to which several clinical characteristics influence them to initiate a patient on parenteral prostacyclin therapy (at any risk level), HCPs indicated that a WHO/NYHA FC of IV or classification of high risk had the most influence (58% and 49% rated as “great deal of influence,” respectively). Patient demographics and characteristics such as cost concerns, health insurance coverage, age, education level, geographic distance from office/clinic, having a good support system, patient concern about site pain, and having a history of drug use were less influential overall, only having a great deal of influence for at most 25% of the HCPs surveyed.

According to HCPs, the main reasons for escalating therapy in patients at intermediate-high risk to parenteral prostacyclin therapy were “patient is declining on their current therapy at the 3-month (or 6-month) follow-up visit” (59% and 58%, respectively), “patient is not improving on their current therapy” (53%), and “patient has right heart hemodynamics consistent with higher risk” (47%). HCPs cited “patients refuse treatment” (51%) and “patient is improving on current therapy” (49%) as the primary reasons for not escalating patients at intermediate-high risk to parenteral prostacyclin therapy. Reasons for escalating/not escalating patients at intermediate-low risk to parenteral prostacyclin therapy were similar.

Upon exploration of patient receptivity to parenteral prostacyclin therapy, most HCPs participating in the qualitative interviews (*n* = 8) reported that their patients are receptive to this treatment, but some (*n* = 4) indicated the opposite. However, HCPs with less-receptive patients primarily saw low-risk or intermediate-risk patients. Most HCPs interviewed (*n* = 11) believed patients start parenteral prostacyclin therapy because their symptoms are progressing quickly and their condition is worsening, essentially leaving patients with “no choice” but to start therapy. HCPs believed that inconvenience of and unfamiliarity with intravenous (IV)/subcutaneous (SC) administration were the main reasons patients resist initiation of parenteral prostacyclin therapy. However, HCPs also indicated that very few patients refuse parenteral prostacyclin therapy once their symptoms progress to a point that they severely impact their quality of life.

**Fig. 2:**
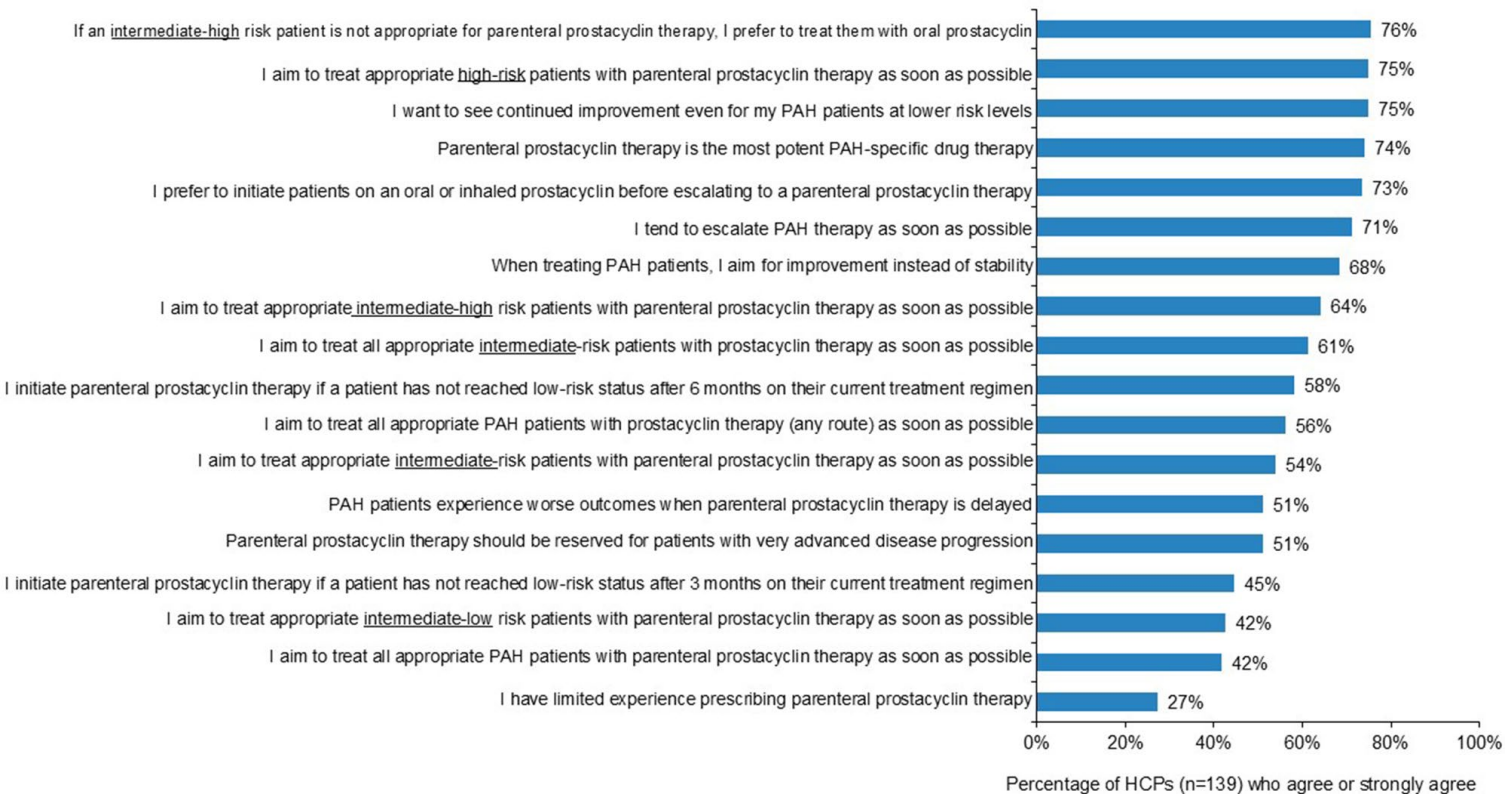
Healthcare professionals’ attitudes towards parenteral prostacyclin therapy. Note: Rated using a 5-point scale where one indicates “strongly disagree” and five indicates “strongly agree.” Abbreviations: HCP, healthcare provider; PAH, pulmonary arterial hypertension

Although most HCPs surveyed “prefer to initiate patients on an oral or inhaled prostacyclin before escalating to a parenteral therapy,” 75% of HCPs reported they “aim to treat appropriate high-risk patients with parenteral prostacyclin therapy as soon as possible” (Fig. 2). Three-quarters of HCPs indicated that they “want to see continued improvement even for [their] PAH patients at lower risk levels” (Fig. 2).

#### Parenteral prostacyclin therapy dosing, documentation, and visit timing

Due to inconsistencies in the dosing information entered in the patient chart forms, we explored how parenteral prostacyclin therapy was typically dosed, titrated, and recorded in EHRs, as well as frequency of follow-up visits. HCPs participating in the qualitative interviews reported that on average, 40% of their patients are initiated on a parenteral prostacyclin therapy in an inpatient setting, and 60% in an outpatient setting. Many HCPs (*n* = 10) reported initiating patients in both inpatient and outpatient settings. HCPs indicated that the target doses they set for their patients depend on a variety of factors including worsening of symptoms/condition, tolerability/side effects, and disease severity. According to the HCPs interviewed, titration approaches are largely based on mode of parenteral administration (IV vs. SC) and setting (inpatient/outpatient). Over half of HCPs (*n* = 8) reported that they typically see their patients for the initial follow-up visit within two weeks of starting parenteral prostacyclin therapy. Most (*n* = 12) see patients for their comprehensive follow-up visit within three months of parenteral prostacyclin therapy initiation.

When asked how PAH treatment is documented in patients’ medical records, HCPs interviewed reported that EHR information is entered by all members of the care team, including physicians and nurses managing titration (inpatient and outpatient). Nearly all HCPs (*n* = 14) indicated that dosing changes are recorded in the EHR, but the location of that information varies based on the type of EHR system the hospital uses. Some HCPs stated that they record dosing and titration information in the notes section, while others record this information in the medication reconciliation section.

### PAH chart review

#### Patient characteristics

A total of 350 patient records were provided by participating HCPs. The average age was 54 years, and the sample was almost evenly split between males and females. Patient record characteristics are shown in Table 2. The most common etiology was idiopathic PAH (50%), followed by PAH associated with connective tissue disease (23%). At diagnosis, most patients were WHO/NYHA FC II or III. Across the study population, the median time from diagnosis to the chart data extraction was 3.8 years.

**Table 2:** Characteristics of intermediate-risk PAH patient records

Characteristics	Patient records (*N* = 350)
Age, mean ± SD	54.1 ± 15.3
Sex, n (%)	
Female	182 (52%)
Male	166 (47%)
Other	2 (1%)
Race/ethnicity^a^, n (%)	
Caucasian or White	202 (58%)
African American or Black	85 (24%)
Hispanic/Latino	26 (7%)
Asian or South Asian	25 (7%)
Other	14 (4%)
Affiliation^b^, n (%)	
PH center	165 (47%)
Community-based	185 (53%)
Specialty^b^, n (%)	
Cardiology	194 (55%)
Pulmonology	114 (33%)
Rheumatology	42 (12%)
Number of years since PAH diagnosis, mean ± SD	4.3 ± 2.0
PAH etiology^c^, n (%)	
Idiopathic	175 (50%)
PAH associated with connective tissue disease	79 (23%)
PAH associated with congenital heart disease	25 (7%)
Heritable	23 (7%)
PAH associated with HIV infection	16 (5%)
Drug- and toxin-induced	14 (4%)
PAH associated with other conditions	11 (3%)
PAH associated with portal hypertension	7 (2%)
Comorbidities^a^	
Hypertension	117 (33%)
Obesity	68 (19%)
Autoimmune disorders	66 (19%)
Hyperlipidemia	63 (18%)
Depression	62 (18%)
Asthma	58 (17%)
Anemia	55 (16%)
Coronary artery disease	55 (16%)
Chronic obstructive pulmonary disease	53 (15%)
Sleep apnea	50 (14%)
Type 2 diabetes	46 (13%)
Thyroid disease	27 (8%)
Interstitial lung disease	23 (7%)
Other condition	80 (23%)
None	46 (13%)
WHO/NYHA FC at diagnosis, n (%)	
I	26 (7%)
II	136 (39%)
III	176 (50%)
IV	8 (2%)
Unknown/not recorded	4 (1%)

^a^ Multiple responses allowed

^b^ Of the healthcare professionals providing the patient record data

^c^ Indicates numbers may not add to 100 due to rounding

Note: PAH etiology response option of “PAH associated with other conditions” and comorbidities option of “other conditions” not specified or defined in the survey

Abbreviations: FC, functional class; HIV, human immunodeficiency virus; PAH, pulmonary arterial hypertension; PH, pulmonary hypertension; SD, standard deviation; NYHA, New York Heart Association; WHO, World Health Organization

#### Visit timing and parenteral prostacyclin therapy use

The median time between the index visit (intermediate risk classification) and parenteral prostacyclin initiation (*n* = 323 patient records with available data) was 4.0 months (IQR: 1.0, 14.0). Between parenteral prostacyclin initiation and the follow-up visit (*n* = 313), the median duration was 3.0 months (IQR: 2.0, 7.0). Following their intermediate risk classification, 53% of patients were initiated on SC treprostinil, 25% on IV treprostinil, and 21% on IV epoprostenol. Over half (55%, *n* = 192) of patients on parenteral prostacyclin therapy did not transition from another prostacyclin; among those who did, 35% (*n* = 55) were previously taking oral treprostinil, 28% (*n* = 44) inhaled treprostinil, 26% (*n* = 41) oral selexipag, and 12% (*n* = 19) inhaled iloprost. HCPs indicated that the main reasons (unaided) for initiating patients on parenteral prostacyclin therapy were clinical worsening/progression (26%), symptoms (17%), efficacy (15%), lack of improvement on prior therapy (11%), risk status (9%), and functional class (8%).

#### Change in risk status and clinical characteristics

**Fig. 3:**
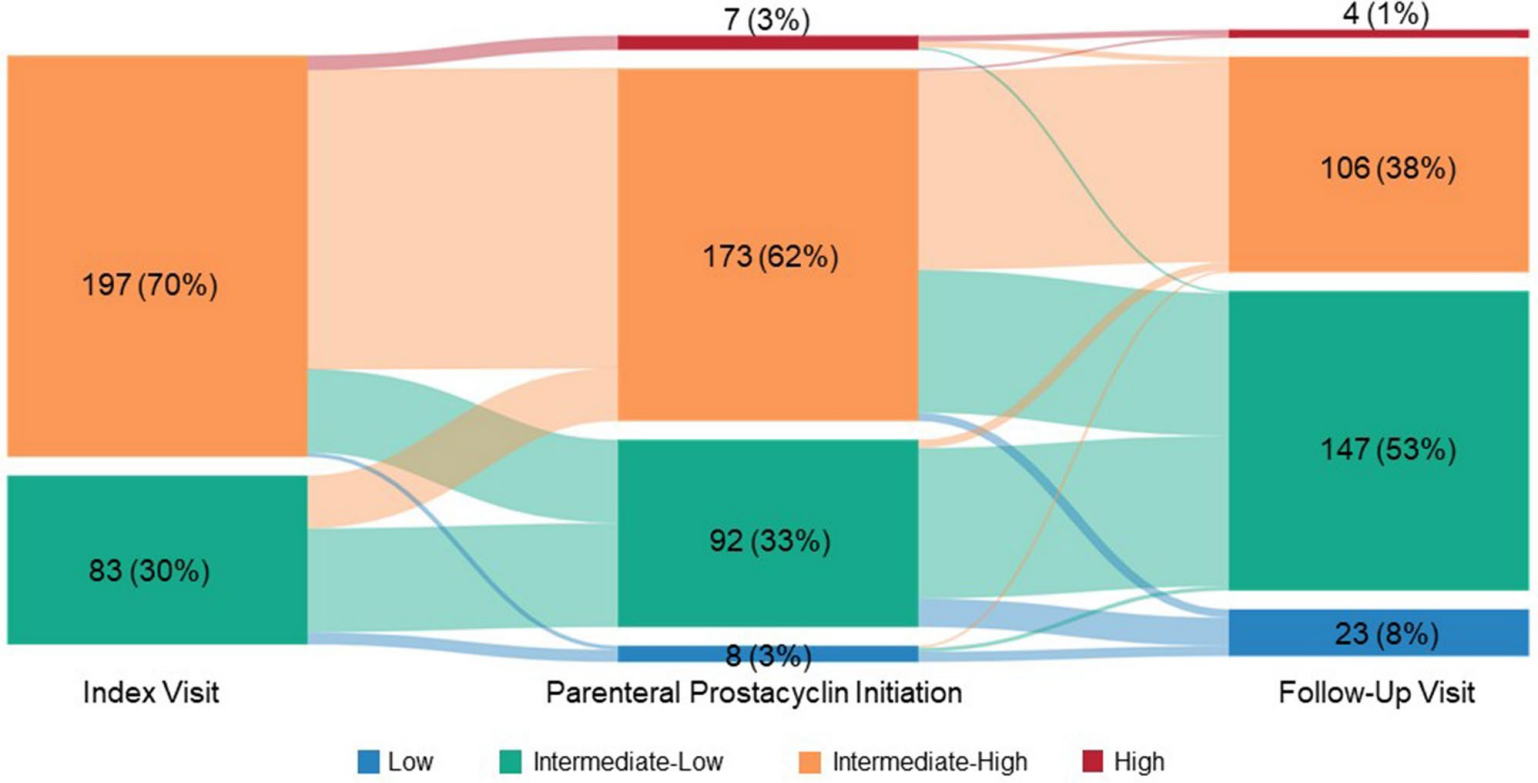
COMPERA 2.0 risk status at index, parenteral prostacyclin initiation, and follow-up (patient record data). Note: PAH Patient Records with all COMPERA 2.0 parameters available at index visit, parenteral prostacyclin initiation, and follow-up visit (*n* = 280). The boxes represent the proportion of patients at each timepoint, and the arcs represent the change between each timepoint; their width is proportional to the number of patients shifting between risk status categories. Index visit indicates timing of intermediate-risk assessment. Abbreviations: COMPERA, Comparative, Prospective Registry of Newly Initiated Therapies for Pulmonary Hypertension; PAH, pulmonary arterial hypertension

At the index visit, the majority of patients (70%) were classified as intermediate-high risk per COMPERA 2.0 risk assessment (Fig. 3). Risk status at index was also calculated using the REVEAL Lite 2 and REVEAL 2.0 risk calculators using available data from the patient records. Among the patient records for which the required parameters were provided in the chart review, 11% were considered low risk, 36% intermediate risk, and 53% high risk, per REVEAL Lite (*n* = 333); 11% were considered low risk, 27% intermediate risk, and 62% high risk, per the REVEAL 2.0 risk calculator (*n* = 338).

Risk status improvements were seen across all three risk calculators. Using REVEAL Lite 2 and REVEAL 2.0 risk assessments at the follow-up visit, 29%/32%, 40%/29%, and 31%/39% were considered low risk, intermediate risk, and high risk, respectively. Per COMPERA 2.0, risk status improved after initiating a parenteral prostacyclin therapy, with 61% of patients at intermediate-low or low risk at their follow-up visit (Fig. 3). Improvements in the individual COMPERA 2.0 risk calculator parameters were seen between parenteral prostacyclin therapy initiation and follow-up, as shown in Table 3. Table 4 presents the clinical characteristics of the patient records at index and follow-up visits. Improvements were seen in disease progression, heart rate, mean right atrial pressure, stroke volume, pericardial effusion, right ventricular function, systolic pulmonary arterial pressure, tricuspid regurgitation severity, and evidence of systolic interventricular septal flattening.

**Table 3:** COMPERA 2.0 parameters at index, parenteral prostacyclin initiation, and follow-up (patient record data)

Parameters	At index visit	At parenteral prostacyclin initiation	At follow-up
WHO/NYHA FC, n (%)	*n* = 350	*n* = 348	*n* = 350
I	25 (7%)	24 (7%)	29 (8%)
II	133 (38%)	95 (27%)	188 (54%)
III	189 (54%)	207 (59%)	121 (35%)
IV	3 (1%)	22 (6%)	12 (3%)
6MWD, median (m) (IQR)	*n* = 350	*n* = 289	*n* = 346
	250.0 (125.0, 332.0)	225.0 (120.0, 300.0)	300.0 (160.0, 380.0)
BNP or NT-proBNP, median (ng/L) (IQR)			
	*n* = 269	*n* = 222	*n* = 268
BNP	215.0 (90.0, 450.0)	209.5 (95.0, 420.0)	152.3 (71.3, 280.0)
	*n* = 161	*n* = 57	*n* = 101
NT-proBNP	500.0 (200.0, 735.0)	680.0 (432.0, 855.0)	500.0 (200.0, 690.0)

Abbreviations: 6MWD, 6-minute walk distance; BNP, brain natriuretic peptide; FC, functional class; IQR, interquartile range; NT-proBNP, N-terminal pro B-type natriuretic peptide; NYHA, New York Heart Association; WHO, World Health Organization

**Table 4:** Clinical characteristics at index and follow-up (patient record data)

Characteristics (index / follow-up)^a^	At index visit (patient records)	At follow-up (patient records)
Disease progression, n (%)	*n* = 332	*n* = 340
Unchanged	96 (29%)	60 (18%)
Improving	85 (26%)	245 (72%)
Declining	151 (45%)	35 (10%)
Heart rate	*n* = 327	*n* = 325
BPM > 96	158 (48%)	77 (24%)
BPM ≤ 96	169 (52%)	248 (76%)
Systolic blood pressure,	*n* = 322	*n* = 321
≥ 110 mmHg	203 (63%)	209 (65%)
< 110 mmHg	119 (37%)	112 (35%)
eGFR < 60mL/min/1.73m^2^ or renal insufficiency, n (%)	*n* = 290	*n* = 283
	50 (17%)	59 (21%)
Predicted DLCO	*n* = 251	*n* = 222
< 40	83 (33%)	66 (30%)
≥ 40	168 (67%)	156 (70%)
Hemodynamic parameters		
PVR (Woods units), median (IQR)	*n* = 123	*n* = 28
	5.0 (4.0, 7.0)	4.0 (3.0, 6.8)
PVR (dynes/sec/cm^− 5^), median (IQR)	*n* = 39	*n* = 9
	400.0 (85.0, 480.0)	100.0 (51.0, 300.0)
mPAP (mmHg), median (IQR)	*n* = 200	*n* = 43
	45.5 (36.0, 56.0)	43.0 (35.0, 56.0)
mRAP (mmHg)	*n* = 205	*n* = 53
> 20 mmHg	126 (61%)	26 (49%)
≤ 20 mmHg	79 (39%)	27 (51%)
Cardiac index (L/min/m^2^), median (IQR)	*n* = 131	*n* = 30
	2.5 (2.0, 3.0)	2.7 (2.3, 4.0)
Cardiac output (thermodilution measurement L/min), median (IQR)	*n* = 71	*n* = 13
	4.1 (3.9, 5.0)	5.4 (4.2, 7.5)
Cardiac output (Fick measurement L/min), median (IQR)	*n* = 42	*n* = 9
	4.5 (4.0, 4.7)	4.1 (4.0, 4.6)
Stroke volume (mL), median (IQR)	*n* = 51	*n* = 9
	50.0 (40.0, 65.0)	58.0 (50.0, 80.0)
Echocardiogram parameters		
Pericardial effusion, n %	*n* = 281	*n* = 152
None	152 (54%)	95 (63%)
Minimal	103 (37%)	47 (31%)
Moderate or large	26 (9%)	10 (7%)
RV function, n %	*n* = 283	*n* = 154
Normal	29 (10%)	16 (10%)
Mildly reduced	113 (40%)	80 (52%)
Moderately reduced	119 (42%)	51 (33%)
Severely reduced	22 (8%)	7 (5%)
RA size, n %	*n* = 283	*n* = 153
Normal	23 (8%)	16 (10%)
Mildly enlarged	118 (42%)	64 (42%)
Moderately enlarged	119 (42%)	67 (44%)
Severely enlarged	23 (8%)	6 (4%)
RV size, n %	*n* = 281	*n* = 153
Normal	25 (9%)	13 (8%)
Mildly enlarged	110 (39%)	73 (48%)
Moderately enlarged	118 (42%)	57 (37%)
Severely enlarged	28 (10%)	10 (7%)
TAPSE (mm), median (IQR)	*n* = 69	*n* = 34
	11.0 (2.0, 16.0)	15.0 (3.0, 17.0)
sPAP (mmHg), median (IQR)	*n* = 184	*n* = 104
	57.5 (50.0, 66.5)	40 (48.0, 56.0)
TR severity	*n* = 256	*n* = 142
None	7 (3%)	7 (5%)
Mild	73 (29%)	59 (42%)
Moderate	155 (61%)	68 (48%)
Severe	21 (8%)	8 (6%)
Did the echocardiogram show systolic interventricular septal flattening?	*n* = 246	*n* = 143
Yes	108 (44%)	39 (27%)
No	138 (56%)	104 (73%)

^a^ Sample sizes reflect the number of patient records where information was known/provided

Abbreviations: BPM, beats per minute; DLCO, diffusing capacity of the lungs for carbon monoxide; eGFR, estimated glomerular filtration rate; IQR, interquartile range; mPAP, mean pulmonary artery pressure; mRAP, mean right atrial pressure; PVR, pulmonary vascular resistance; RA, right atrial; RV, right ventricular; SD, standard deviation; sPAP, systolic pulmonary arterial pressure; TAPSE, tricuspid annular plane systolic excursion; TR, tricuspid regurgitation

## Discussion

This study aimed to gain a better understanding of parenteral prostacyclin therapy use in the intermediate-risk PAH population in a real-world setting. In our patient chart review study, 50% of patients had idiopathic PAH, similar to that seen in REVEAL and in a global physician survey and chart review study [21, 26]. Slightly more than half of patients were female, less than that seen in other studies of patients with PAH [11, 27–29]. Patients were primarily WHO/NYHA FC II or III at diagnosis, similar to that observed in other research [11, 21, 28, 29]. The affiliation of the treating HCP was almost evenly split between PH centers and community-based institutions.

We found that a median of 4 months passed between the index visit of intermediate-risk classification and initiation of parenteral prostacyclin therapy, with this route of administration being the first prostacyclin therapy for the majority of patients. Worsening of PAH symptoms was the primary reason HCPs cited for initiating patients on parenteral prostacyclin therapy. Patients in our analysis showed improvement in risk status according to the COMPERA 2.0 risk assessment between parenteral prostacyclin therapy initiation and the follow-up visit, which occurred six months later, on average. Approximately two-thirds of patients (for whom all COMPERA 2.0 parameters available were available at index visit, parenteral prostacyclin initiation, and follow-up visit) were at intermediate-high risk at treatment initiation, decreasing to 38% at follow-up. Our findings are similar to improvements seen in the French PH Registry between diagnosis and first re-evaluation (median of 4.4 months) among patients enrolled between 2006 and 2016 taking monotherapy or combination PAH targeted therapy [30] and a database cohort analysis of patients diagnosed with idiopathic PAH between 1999 and 2009 at three- and 12-month follow-up [31]. 

Parenteral prostacyclin therapy has been shown to have positive effects on invasive hemodynamic measures, which we observed across several parameters in our real-world research. A single-site study conducted between 2007 and 2016 among patients with PAH initiating IV epoprostenol or IV or SC treprostinil therapy showed that early initiation of parenteral prostacyclin therapy was associated with decreases in hemodynamic measures including mean pulmonary artery pressure (mPAP) and pulmonary vascular resistance [32]. In a retrospective pilot study of patients newly diagnosed with PAH, mPAP was reduced after four months of treatment with upfront triple combination therapy with IV epoprostenol [33]. In high-risk patients with PAH, upfront triple therapy with SC treprostinil was associated with improvements in clinical and hemodynamic parameters and right heart reverse remodeling [34]. Boucly et al. found that triple combination therapy with parenteral prostacyclin was associated with a significantly lower risk of death in intermediate-risk patients as compared with dual combination therapy or monotherapy [35]. 

We found that the dosing information collected in the patient chart records were varied and inconsistent. One of our assumptions was that dosing may be dependent on the location of parenteral prostacyclin therapy initiation or route of administration (e.g., patients taking SC treprostinil may have been switched from IV administration after hospital discharge); however, information about where patients received their first dose of parenteral prostacyclin therapy (i.e., inpatient or outpatient) was not collected in our study. In the qualitative interviews, we found that approaches to parenteral prostacyclin therapy initiation and titration varied by patient and by treating HCP. It is also possible that dosing changes may not be reflected in real-time in some EHRs and instead may be reflected elsewhere in the patient’s chart, which was supported by the qualitative interviews. Additionally, as parenteral prostacyclin dosing is calculated based on patient weight to provide a flow rate specific to a pump, there are several pieces of information that may be documented. Lastly, the drug order in the EHR may not be accurate/adjusted and may be documented in the notes under “medication reconciliation.” Although the survey and chart review form were pilot tested, it is possible that HCPs were uncertain as to how to record the dosing information.

The demographics of our HCP survey were similar to that of another recent survey of clinicians evaluating the use of risk assessment tools in PAH management; [36] however, our study had a greater representation of cardiologists and those practicing at a PH center. HCPs self-reported performing risk assessments in less than 60% of their PAH patient visits, with the COMPERA 2.0 risk status method rarely used. HCPs reported relying on gestalt/their clinical impression 19% of the time, particularly those in community-based practices. Despite ESC/ERS Guidelines since 2015 recommending the use of risk stratification methods to manage PAH [23] and shown superiority over physician gestalt [37], low use of risk assessment tools has been reported in the literature [36, 38]. In our study, reasons for not conducting formal risk assessments at every PAH visit were patient stability, performing informal assessments, and the time-intensiveness of formal assessments.

The “paradox” of delaying or underutilizing parenteral prostacyclin therapy despite the known survival benefits of parenteral prostacyclin therapy discussed in Schilz et al. were also observed in our survey results [39]. Improvement in survival and clinical efficacy were selected by HCPs as the most important attributes when prescribing therapy to patients with PAH at intermediate risk. On the contrary, FC IV or high-risk status most influenced HCPs to initiate patients on parenteral prostacyclin therapy. Respondents escalated patients at intermediate-high risk to parenteral prostacyclin therapy due to various factors including lack of improvement or progression and right heart hemodynamics. Most HCPs (75%) reported aiming to treat appropriate high-risk patients with parenteral prostacyclin therapy as soon as possible, but fewer agreed regarding their patients at intermediate-high (64%), intermediate (54%), or intermediate-low risk (42%).

Only half of HCPs agreed that patients experienced worse outcomes when parenteral therapy is delayed. However, treatment delays can negatively impact survival [40]. Earlier initiation may positively affect prognosis as seen in two small studies [33, 34]. Additionally, an analysis of patients enrolled in the French PH Registry initiated on PAH targeted medications within three months of PAH diagnosis demonstrated that initial triple combination therapy including a parenteral prostacyclin was associated with a higher rate of survival [35]. Potential barriers to prescribing parenteral prostacyclin therapy may include patient age, comorbidities, patient discomfort with pump therapy, geographic distance from the office/clinic/PH center, concerns about overall medication compliance, lack of an appropriate support system, and history of drug use [41–43]; however, we found that patient demographics and characteristics were least influential in initiating parenteral prostacyclin therapy according to the HCPs surveyed.

In our survey, HCPs cited patient refusal as one of the key reasons for not escalating patients at intermediate-high or intermediate-low risk to parenteral prostacyclin therapy. Patients may refuse this therapy option for a variety of reasons including fear of negative impact on quality of life, administering the medication, challenges with having to wear an external pump, and site pain/infections [44]. There may also be healthcare and provider barriers such as limitations of risk assessment tools (e.g., lack of right ventricular assessment variables, for which most other components are surrogates), access to care, lack of infrastructure or nursing staff to support parenteral prostacyclin therapy in the community care setting, suboptimal communication when recommending the treatment option to patients, infrequently performing risk assessments, and lack of knowledge or experience with parenteral prostacyclin therapy.

Although prescribing PAH targeted treatment at initial diagnosis is relatively straightforward following current clinical guidelines, HCPs face challenges in deciding when and how to escalate treatment for patients on therapy. Overcoming barriers to earlier initiation of parenteral prostacyclin therapy will likely take a multifaceted approach including encouraging earlier referral to PH centers [45], increasing education about echocardiogram and other right heart parameters that can inform the need for parenteral prostacyclin therapy, education on how to evaluate echocardiogram parameters over time, and using patient/nurse videos to educate patients and instill confidence in their ability to manage parenteral prostacyclin therapy. Additional research could provide evidence for setting higher expectations for specific treatment goals in the first three to six months. Prospective randomized studies are also needed to determine extended survival as seen in recent research.

## Limitations

Assessing HCP perceptions of PAH management and its treatment with a chart review allows us to contextualize attitudes and behaviors with practice patterns, including those of HCPs who are community-based. We followed best practices for chart audit studies discussed in Vassar et al. [46], including creating well-defined, clearly articulated research questions, considering sampling issues a priori, using standardized data collection forms, explicitly specifying inclusion and exclusion criteria, and conducting pilot testing; however, this study has limitations. These include the retrospective nature of the study, potential selection bias of patient chart records, recall bias, and the accuracy of the patient records. As a retrospective chart review, the study is limited to describing the associations between treatment and outcomes rather than causality. Potential confounding variables could affect patient outcomes, including concomitant therapies or variations in clinical practice. The majority of our survey respondents were cardiologists who have a baseline standard training in echocardiography and right heart catheterization; thus, practice patterns described in our study may not be generalizable to other HCPs treating patients with PAH. Additionally, the demographic characteristics of the HCPs who responded to the survey and provided patient records may differ from those who did not respond to and complete the survey. The duration of follow-up captured in the chart review was brief (median of 3.0 months), which limits our ability to understand long-term outcomes and durability of treatment effects. Further, the study was sponsored by a pharmaceutical company that develops treatments for PAH; however, the study sponsor was blinded to the study respondents; clinical experts were involved in the design of the study, survey, and chart review form; and a third party conducted the data collection and analyzed the results. The inability to analyze the parenteral prostacyclin dosing for the patient records is also a limitation and prevents us from making any potential associations regarding dosing and outcomes. However, even with a short follow-up period, improvements were observed in patients’ risk status and clinical parameters between treatment initiation and first comprehensive follow-up. Further research is needed to confirm these findings and refine treatment strategies for patients with PAH.

## Conclusions

The HCPs surveyed aim to improve the outcomes of their patients with PAH at lower risk levels and agree that parenteral prostacyclin therapy is effective. According to the chart review, patients’ risk status and clinical and hemodynamic parameters improved between parenteral prostacyclin therapy at follow-up, supporting recent guidelines suggesting earlier use of this treatment in intermediate-risk patients. Additional education and resources are needed to increase awareness of the advantages of using formal risk assessments and the benefits of parenteral prostacyclin therapy. A graphical abstract summarizing this research can be found in Supplementary Material 2.

## Electronic supplementary material

Below is the link to the electronic supplementary material.

Supplementary Material 1

Supplementary Material 2

## Data Availability

The datasets used and/or analyzed during the current study are available from the corresponding author on reasonable request.

## Abbreviations

PAH: Pulmonary arterial hypertension
COMPERA: Comparative, Prospective Registry of Newly Initiated Therapies for Pulmonary Hypertension
HCPs: Healthcare professionals
WHO: World Health Organization
FC: Functional Class
6MWD: 6-minute walk distance
BNP: B-type natriuretic peptide
NT-proBNP: N-terminal pro B-type natriuretic peptide
REVEAL: Registry to Evaluate Early and Long-Term PAH Disease Management
ESC: European Society of Cardiology
ERS: European Respiratory Society
NYHA: New York Heart Association
SD: Standard Deviation
IQR: Interquartile range
EHR: Electronic Health Record
IV: Intravenous
SC: Subcutaneous
mPAP: Mean pulmonary artery pressure
