# Supplementary material for: Parenteral prostacyclin utilization in patients with pulmonary arterial hypertension in the intermediate-risk strata: a retrospective chart review and cross-sectional survey
Source: BMC Pulm Med. 2024 Nov 20;24:574. doi: 10.1186/s12890-024-03388-w (PMC11577822; doi:10.1186/s12890-024-03388-w)
Supplement: Supplementary file 1 — Supplementary Material 1 [file 12890_2024_3388_MOESM1_ESM.pdf]

Title: Real-world assessment of parenteral prostacyclin therapy in patients with intermediate-risk pulmonary arterial hypertension: a retrospective chart review and cross-sectional survey

Authors: Anjali Vaidya, Margaret R. Sketch, Meredith Broderick, Oksana A. Shlobin

Supplemental Material

PAH Therapy Utilization Survey & Chart Audit Form

SECTION S: SCREENER

ALL RESPONDENTS

S1 Thank you for your interest in this survey. We appreciate your willingness to participate in this important research on healthcare issues.

Before participating, KJT Group requires you to review the following information:

- KJT Group is a **global market research company**.
- Your responses to this survey will **help the sponsor design new products/services to meet patient needs**.
- Your participation involves completing this survey and anonymized patient chart forms.
- We expect, on average, it will take respondents like yourself **20 minutes** to complete this survey and **10 minutes** to complete each patient chart form.
- If you qualify, we will ask you to provide anonymized patient charts (more information will be provided if you qualify for the survey).
- Findings of this research project may be published in **scientific journals** or presented at medical meetings. All the results will be presented in aggregate form; data will never be presented in a way that identifies individual institutions.
- Your responses will be kept **strictly confidential** and will never be associated with your name.
- Your **participation is voluntary,** and you may choose to stop participating at any time (withdraw consent).

If you qualify and complete this research, you will be eligible to receive the honorarium referenced in your invitation.

Do you consent to these terms and wish to continue with the survey?

1. Yes [CONTINUE]
2. No [TERMINATE]

[IF CONSENT (S1r1) CONTINUE. ELSE TERMINATE.]

CONSENTS (S1r1)

AE1 We are required to pass on to our client, who is a pharmaceutical company, details of adverse events related to their product(s), mentioned during the course of this research. Although your responses will be treated in confidence, we are required to report any mention of an adverse event for a specific patient, even if it has already been reported to the company or the FDA. In such a situation, you will be asked whether or not you are willing to waive the confidentiality given to you under the Market Research Codes of Conduct specifically in relation to that adverse event. Everything else you share in this survey will continue to remain confidential, and you will still have the option to remain anonymous if you wish.

By proceeding to the next screen, you confirm that you have read, understood and accept the points above and are happy to proceed with the research survey on this basis.

Are you happy to participate in the survey based on this information?

1. Yes [CONTINUE]
2. No [TERMINATE]

[IF AGREES TO AE REPORTING (AE1r1) CONTINUE. ELSE TERMINATE.]

AGREES TO AE REPORTING (AEr1)

S2 Which of the following best describes your professional title?

[ALPHA SORT]

1. Physician (MD or DO)
2. Nurse Practitioner (NP)
3. Physician Assistant (PA)
4. Advanced Practice Registered Nurse (APRN)
5. Registered Nurse (RN)
6. Medical Assistant
7. Non-provider (Discharge coordinator, social worker, administrative, etc.) [ANCHOR]
8. Other [ANCHOR]

[IF PHYSICIAN, NP, OR PA, OR APRN (S2r1-4), CONTINUE. ELSE TERMINATE.]

PHYSICIAN, NP, PA, OR APRN (S2r1-4)

S3 Which of the following is your primary medical specialty?

[ALPHA SORT]

1. Pulmonology
2. Cardiology
3. Rheumatology
4. General practice/Family medicine/Internal medicine
5. Hospitalist
6. Surgery
7. Critical Care
8. Other, please specify: [MANDATORY TEXT BOX] [ANCHOR]

[IF PULMONOLOGY, CARDIOLOGY, RHEUMATOLOGY (S3r1-3), CONTINUE. ELSE MARK UNQUALIFIED AND CONTINUE.]

PHYSICIAN, NP, PA, OR APRN (S2r1-4)
S5 How many years have you been in practice [IF PHYSICIAN (S2r1) INSERT "beyond your residency or fellowship"]?

[IF PHYSICIAN (S2r1) INSERT "*If you are still in your residency or are currently a fellow, please indicate below.*"] *If you have not been in practice for at least one year, please enter "0" (zero).*

[RANGE: 0-50]
1. |_|_| year(s) in practice
99. Still in residency, or currently a fellow [SHOW FOR PHYSICIAN ONLY (S2r1)]

[IF IN ROLE FOR 2+ YEARS (S5>1) CONTINUE. ELSE MARK UNQUALIFIED AND CONTINUE]

PHYSICIAN, NP, PA, OR APRN (S2r1-4)
S6 In what state is your practice located?

If your institution is location in more than one state, please select the state where the facility you primarily practice is located.

[INSERT DROP DOWN MENU IN ALPHA ORDER]

[IF PRACTICES IN US AND NOT VT (S6/NE VT, 99) CONTINUE. ELSE TERMINATE]

PRACTICES IN U.S. AND NOT VT (S6/NE VT, 99)
S6B HIDDEN QUESTION FOR REGION

1. Northeast
   [S6=CT, MA, ME, NH, NJ, NY, PA, RI, VT]
2. Midwest
   [S6=IA, IL, IN, KS, MI, MN, MO, ND, NE, OH, SD, WI]
3. South
   [S6=AL, AR, DC, DE, FL, GA, KY, LA, MD, MS, NC, OK, SC, TN, TX, VA, WV]
4. West
   [S6=AK, AZ, CA, CO, HI, ID, MT, NM, NV, OR, UT, WA, WY]

PRACTICES IN U.S. AND NOT VT (S6/NE VT, 99)

S8 In the past 12 months, how many patients did you personally manage or treat for **WHO Group 1 Pulmonary Arterial Hypertension (PAH)**?

For reference, the following are classified as WHO Group 1 Pulmonary Arterial Hypertension (PAH):<br><br>• Idiopathic (IPAH)<br>• Heritable (HPAH)<br>o Bone morphogenetic protein receptor type 2 (BMPR2) gene mutations<br>o Activin receptor-like kinase 1 (ALK1) or endoglin gene mutations (with or without hemorrhagic telangiectasia)<br>o Unknown<br>• Drug- and toxin-induced<br>• Associated with (APAH):<br>o Connective tissue diseases<br>o Human immunodeficiency virus (HIV) infection<br>o Portal hypertension<br>o Congenital heart disease (CHD)<br>o Schistosomiasis<br>o Chronic hemolytic anemia<br>• Persistent pulmonary hypertension of the newborn (PPHN)

[RANGE: 0-999]

1. |_|_|_| WHO Group 1 PAH patients personally managed or treated in past 12 months

[IF HAS 20+ PAH PATIENTS (S8r1>19), CONTINUE. IF HAS <20 PAH PATIENTS (S8r1/0-19), MARK UNQUALIFIED AND CONTINUE. ELSE TERMINATE]

HAS PAH PATIENTS (S8r1>0)

S10 Of the [INSERT S8r1] **PAH (WHO Group 1)** patients you have managed or treated in the past 12 months, for how many did you personally initiate a PAH treatment or oversee the initiation of a PAH treatment?

By "initiate" we mean either initiating a new prescription or switching/adding another PAH medication to a treatment regimen. Please do not count dose adjustments.

[RANGE: 0-S8r1]

1. |_|_|_| PAH (WHO Group 1) patients initiated on treatment

[IF INITIATED TREATMENT FOR AT LEAST 10 PAH PATIENTS (S10>9) CONTINUE. IF LESS THAN 10 PATIENTS (S10<10) MARK UNQUALIFIED AND CONTINUE. ELSE TERMINATE]

INITIATES PAH TREATMENT (S10>0)
S11 Which of the following types of PAH medications do you personally **currently** initiate for PAH patients?

Please select all that apply.
[MULTI-SELECT; RANDOMIZE, GROUP R3-4]
1. Oral (PO)
2. Inhaled
3. Subcutaneous (SUBCUT, SC, SQ)
4. Intravenous (IV)
5. None of the above [EXCLUSIVE; ANCHOR]

[IF INITIATES WITH PARENTERAL THERAPY (S11r3-4) CONTINUE. ELSE MARK UNQUALIFIED AND CONTINUE]

INITIATES ORAL TREATMENT (S11r1)
S11A You indicated that you personally initiate or manage oral medications for your PAH patients. Which following types of oral medications do you currently initiate for patients?

Please select all that apply.
[MULTI-SELECT; RANDOMIZE]

1. ERAs
2. PDE5 inhibitors
3. sGC stimulators
4. Prostanoids/IP receptor agonists
5. None of the above [EXCLUSIVE; ANCHOR]

HAS PAH PATIENTS (S8r1>0)
S9 Among the **PAH (WHO Group 1)** patients you have personally managed or treated in the past 12 months, based on your clinical judgement, what proportion of these patients would you **currently** classify under each of the following classifications for risk of death in one year?

Your best estimate will do. Your responses must sum to 100%.

[RANGE: 0-100 FOR EACH ROW, MUST SUM TO 100, SHOW CONSTANT SUM INDICATOR]

1. Low risk |_|_|_| % of patients
2. Intermediate risk |_|_|_| % of patients
3. High risk |_|_|_| % of patients

HAS PAH PATIENTS (S8r1>0)
S9B HIDDEN QUESTION FOR NUMBER OF INTERMEDIATE RISK PATIENTS

[CALCULATE S9*S8 FOR EACH ROW. ROUND TO NEAREST WHOLE NUMBER]

1. Low Risk |_|_|_|
2. Intermediate risk |_|_|_|
3. High risk |_|_|_|

[IF (S9Br2>4) CONTINUE, ELSE MARK UNQUALFIED AND CONTINUE]

HAS PAH PATIENTS (S8r1>0)
S12 Which of the following types of facilities best describes where you currently treat PAH (WHO Group 1) patients?

Please select one.

1. PHA Accredited Center of Comprehensive Care
2. PHA Accredited Regional Care Center
3. Unaccredited University-based Center
4. Unaccredited Community-based Center
5. Private setting
6. Other setting, please specify: [TEXT BOX]

HAS PAH PATIENTS (S8r1>0)
S13 Which of the following statements best describes how you generally manage PAH (WHO Group 1) patients?

1. I personally manage and initiate treatments for all my PAH patients, regardless of their risk status/disease severity
2. I personally manage and initiate treatments for all my low risk PAH patients, but refer intermediate and high risk patients to a PAH specialist or a PAH Center
3. I personally manage and initiate treatments for all my low and intermediate risk PAH patients, but refer high risk or more severe PAH patients to a PAH specialist or a PAH Center
4. PAH patients are often referred to me for PAH treatment or management by other physicians

ALL QUALIFIED RESPONDENTS
S16 Based on your responses, you have met the qualification criteria for this study.

The purpose of this study is to better understand parenteral prostacyclin utilization and impact in intermediate risk patients.

This study consists of two parts:

1. The first part is a survey focusing on PAH patients and treatments.
2. The second part consists of a patient chart exercise where you will be asked to complete patient chart forms for your **patients with PAH classified as intermediate risk and**

subsequently initiated on a <u>parenteral prostacyclin therapy</u>, with at least 1 follow-up visit available assessing WHO Functional Class, 6-minute walking distance, and NT-proBNP/BNP levels.

- We will not ask for patient names, nor identify you or your patients in any way.
- Please be aware that this study is fully HIPAA-compliant.
- All data will be reported for groups, and not individuals.
- For your convenience, the charts can be completed all at once or one at a time. You may leave the survey and come back throughout the time you complete the charts. You may also have another staff member complete the chart audit form.
- You will be asked to complete a minimum of 2 patient charts, with the option of providing up to 10 charts.

Would you like to continue with this research?

1. Yes
2. No

[IF NOT INTERESTED IN CONTINUING (S16r2) TERMINATE]

ALL RESPONDENTS
S100 FINAL QUOTA QUESTION [n=100]

1. **Pulmonologists [n=50]**
   - Consents (S1r1)
   - Agrees to AE reporting (AEr1)
   - Physician or NP/PA/APRN (S2r1-4)
   - Specialty in pulmonology (S3r1)
   - In role for 2+ years (S5>1)
   - Practices in U.S. but not VT (S6/NE VT, 99)
   - Treats at least 20 PAH patients (S8r1>19)
   - Treats at least 5 intermediate risk patients (S9Br2>4)
   - Initiated 10 treatments in past 12 months (S10>9)
   - Initiates parenteral therapy (S11r3-4)
   - Agrees to participate (S16R1)
2. **Cardiologists [n=50]**
   - Consents (S1r1)
   - Agrees to AE reporting (AEr1)
   - Physician or NP/PA/APRN (S2r1-4)
   - Specialty in cardiology (S3r2)
   - In role for 2+ years (S5>1)
   - Practices in U.S. but not VT (S6/NE VT, 99)
   - Treats at least 20 PAH patients (S8r1>19)

CONFIDENTIAL

- Treats at least 5 intermediate risk patients (S9Br2>4)
- Initiated 10 treatments in past 12 months (S10>9)
- Initiates parenteral therapy (S11r3-4)
- Agrees to participate (S16R1)

3. **Rheumatologists** **[n=10]**
    - Consents (S1r1)
    - Agrees to AE reporting (AEr1)
    - Physician or NP/PA/APRN (S2r1-4)
    - Specialty in rheumatology (S3r3)
    - In role for 2+ years (S5>1)
    - Practices in U.S. but not VT (S6/NE VT, 99)
    - Treats at least 20 PAH patients (S8r1>19)
    - Treats at least 5 intermediate risk patients (S9Br2>4)
    - Initiated 10 treatments in past 12 months (S10>9)
    - Initiates parenteral therapy (S11r3-4)
    - Agrees to participate (S16R1)

ALL RESPONDENTS
S105 SOFT QUOTA – CENTER/COMMUNITY

1. PAH Center (S12r1-2) [n=9999]
2. Community-based (S12r3-6) [n=9999]

ALL RESPONDENTS
S110 SOFT QUOTA – ROLE

1. Physician (S2r1) [n=9999]
2. NP/PA/APRN (S2r2-4) [n=9999]

ALL RESPONDENTS
S115 SOFT QUOTA – REGION

1. Northeast (S6Br1) [n=9999]
2. Midwest (S6Br2) [n=9999]
3. South (S6Br3) [n=9999]
4. West (S6Br4) [n=9999]

SECTION 200: PAH PATIENT POPULATION

ALL QUALIFIED RESPONDENTS (S100r1-3)
Q200 You have qualified for the full survey. Thank you for your responses thus far. The remainder of this survey should take approximately 18 minutes to complete. Following completion of the survey, you will receive instructions to begin the patient chart exercise.

As a reminder, your responses to this survey are critical to the success of this research in helping the sponsor design new products/services to meet patient needs. Your responses will be kept strictly confidential and only reported in combination with other respondents' data. In addition, you may be asked certain questions for quality control purposes.

Please click "Continue."

ALL QUALIFIED RESPONDENTS (S100r1-3)
Q201 To begin, among the **PAH (WHO Group 1)** patients that you currently manage, what proportion came to be under your care in the following ways?

Your best estimate will do. Your responses must sum to 100%.

[RANGE 0-100; TOTAL MUST SUM TO 100%; INSERT CONSTANT SUM INDICATOR, AUTOFILL ZEROES]

	% of PAH patients
1. Came directly to your practice and were diagnosed by you	|_|_|_| %
2. Diagnosed by another physician and referred to you for treatment	|_|_|_| %
3. Referred by another physician and then diagnosed and treated by you	|_|_|_| %
4. Referred to you after another physician had diagnosed the patient and initiated treatment	|_|_|_| %

ALL QUALIFIED RESPONDENTS (S100r1-3)
Q202 From which types of specialists do you most commonly receive referrals for **PAH (WHO Group 1)** patients?

Please select all that apply.

[MULTI-SELECT, ALPHA SORT]

1. Internal Medicine
2. General Practice/Family Practice
3. Cardiology
4. Pulmonology
5. Rheumatology
6. Hospitalist
7. Other specialty, please specify: [TEXT BOX] [ANCHOR]
8. I do not receive referrals for PAH patients [EXCLUSIVE] [ANCHOR]

ALL QUALIFIED RESPONDENTS (S100r1-3)
Q210 Now, among the **intermediate risk** patients that you currently manage, what proportion of these PAH patients were classified under each of the following WHO/NYHA Functional Classes **<u>at the time of their diagnosis</u>**?

Your best estimate will do. Your responses must sum 100%.

[RANGE: 0-100 FOR EACH; TOTAL MUST SUM TO 100; INSERT CONSTANT SUM INDICATOR, AUTOFILL ZEROES]

	**% of intermediate risk PAH patients <u>at the time of their diagnosis</u>**
1. Functional Class I	|_|_|_| %
2. Functional Class II	|_|_|_| %
3. Functional Class III	|_|_|_| %
4. Functional Class IV	|_|_|_| %
5. Unknown	|_|_|_| %

ALL QUALIFIED RESPONDENTS (S100r1-3)
Q213 Again, among the **intermediate risk** patients that you currently manage, what proportion of these PAH patients are **<u>currently</u>** classified under each of the following WHO/NYHA Functional Classes?

Your best estimate will do. Your responses must sum 100%.

[RANGE: 0-100 FOR EACH; TOTAL MUST SUM TO 100; INSERT CONSTANT SUM INDICATOR, AUTOFILL ZEROES]

	**% of intermediate risk PAH patients <u>currently</u>**
1. Functional Class I	|_|_|_| %
2. Functional Class II	|_|_|_| %
3. Functional Class III	|_|_|_| %
4. Functional Class IV	|_|_|_| %
5. Unknown	|_|_|_| %

ALL QUALIFIED RESPONDENTS (S100r1-3)
Q215 Among the **intermediate risk** patients that you currently manage, what proportion of these patients would you classify as each of the following?

Your best estimate will do. Your responses must sum to 100%.

[RANGE: 0-100 FOR EACH; TOTAL MUST SUM TO 100; INSERT CONSTANT SUM INDICATOR, AUTOFILL ZEROES]

	**% of intermediate risk PAH patients**
1. Unchanged (*<u>neither</u> improving nor declining since their previous visit*)	|_|_|_| %

2. Improving (*<u>improvement</u> in disease progression since their previous visit*) |_|_|_| %
3. Declining (*<u>decline</u> in disease progression since their previous visit*) |_|_|_| %

SECTION 300: PAH DIAGNOSIS, RISK ASSESSMENT, & MONITORING

ALL QUALIFIED RESPONDENTS (S100r1-3)
Q310 <u>On average</u> among your total PAH patient population, how often do you use the following methods to assess your patients' <u>response to their PAH-specific drug therapy</u>?

Please select one answer in each row.

[COLUMNS]

1. Every 3 months
2. Every 6 months
3. Every 12 months
4. Less often than every 12 months
5. I never use this assessment with my patients

[ROWS, RANDOMIZE]
1. Vital signs (e.g., blood pressure, heart rate, pulse oximetry)
2. 6-minute walking test (6MWT)
3. Assessment of WHO/NYHA Functional Class
4. Echocardiography
5. Electrocardiogram
6. Cardio-pulmonary exercise testing
7. Right heart catheterization
8. NT-proBNP/BNP levels
9. Diffusing capacity for carbon monoxide (DLCO)
10. eGFR or evaluation for renal insufficiency
11. Cardiac MRI
12. Spirometry
13. For quality control purposes, please select 'Every 12 months'
14. Other, please specify: [ANCHOR,TEXT BOX]

ALL QUALIFIED RESPONDENTS (S100r1-3)
Q320 We'd now like to understand more about your use of <u>formal risk assessments</u>.

In what proportion of your <u>patient visits</u> do you perform a <u>formal risk assessment</u>?

If you do not perform formal risk assessments with any of your patients, please enter "0" (zero).

[RANGE 0-100]
1. |_|_|_| % of visits in which a formal risk assessment is performed

DOES NOT CONDUCT FORMAL RISK ASSESSMENT (Q320r1<100)
Q320B For what reasons do you not conduct formal risk assessments at every visit with your PAH patients?

Please be as specific as possible.

[MANDATORY TEXT BOX]

USES FORMAL RISK ASSESSMENT (Q320r1>0)
Q325 When conducting a formal risk assessment, what proportion of the time do you use each of the following formal risk assessment methods?

Your best estimate will do. Your responses must sum to 100%.

[RANDOMIZE, GROUP R2-3, GROUP R5-6, RANGE 0-100, MUST SUM TO 100%, SHOW CONSTANT SUM INDICATOR, AUTOFILL ZEROES]

1. ESC/ERS guidelines |_|_|_| %
2. French Noninvasive Criteria |_|_|_| %
3. French Invasive Criteria |_|_|_| %
4. COMPERA/COMPERA 2.0 |_|_|_| %
5. REVEAL 2.0 |_|_|_| %
6. REVEAL Lite 2 |_|_|_| %
7. Gestalt / Clinical impression (without formal risk calculator) [ANCHOR] |_|_|_| %
8. Other, please specify: [TEXT BOX] [ANCHOR] |_|_|_| %

ALL QUALIFIED RESPONDENTS (S100r1-3)
QC2 For quality control purposes, please select “slightly unhappy” from the list of options below.

1. Very unhappy
2. Slightly unhappy
3. Neutral
4. Slightly happy
5. Very happy

SECTION 400: PAH TREATMENT LANDSCAPE

ALL QUALIFIED RESPONDENTS (S100r1-3)
Q401 [MAX DIFF EXERCISE]
[ON THE FIRST SCREEN: "Thank you for your responses thus far! We would now like you to consider several attributes that might influence your decision when prescribing a therapy for the treatment of PAH to your **intermediate risk patients**. Considering only the attributes listed on the screen, please select the attribute that is most important in your therapy selection and then select the least important attribute."

[ON SUBSEQUENT SCREENS: "Considering only the attributes listed on the screen, please select the attribute that is most important in your therapy selection for **intermediate risk patients** and then select the least important attribute."]

[EXAMPLE SCREEN BELOW; INSERT SCREEN COUNTER AT THE TOP OF EACH SCREEN "1 of 10, etc."]

**Most** Important		**Least** Important
○	Has proven clinical efficacy	○
○	Reduces risk status	○
○	Improves survival	○

1. Has proven clinical efficacy
2. Reduces risk status
3. Improves survival
4. Has good insurance coverage
5. Offers patient assistance programs
6. Has a favorable safety/tolerability profile
7. Familiarity with the product
8. Is widely prescribed for the treatment of PAH
9. Low patient refusal rate
10. High rate of patient compliance
11. Provides dosing flexibility

ALL QUALIFIED RESPONDENTS (S100r1-3)
Q400 We would now like to understand more about your patients being treated with prostacyclin therapy.

Among your **PAH (WHO Group 1)** patients currently being treated with a prostacyclin drug therapy (i.e., oral, inhaled, or parenteral prostacyclin), what was their risk classification prior to starting prostacyclin therapy?

Your best estimate will do. Your responses must sum to 100%.

[RANGE: 0-100 FOR EACH ROW, MUST SUM TO 100%, SHOW CONSTANT SUM, AUTOFILL ZEROES]

	**% of patients treated with prostacyclin drug therapy**
1. Low risk	|_|_|_| %
2. Intermediate risk	|_|_|_| %
3. High risk	|_|_|_| %

ALL QUALIFIED RESPONDENTS (S100r1-3)
Q400D Now, among your **PAH (WHO Group 1)** patients currently being treated with a **parenteral** prostacyclin drug therapy, what was their risk classification prior to starting parenteral prostacyclin therapy?

Your best estimate will do. Your responses must sum to 100%.

[RANGE: 0-100 FOR EACH ROW, MUST SUM TO 100%, SHOW CONSTANT SUM]

	**% of patients treated with parenteral prostacyclin drug therapy**
1. Low risk	|_|_|_| %
2. Intermediate risk	|_|_|_| %
3. High risk	|_|_|_| %

ALL QUALIFIED RESPONDENTS (S100r1-3)
Q416 On average, what proportion of your parenteral prostacyclin patients are initiated on a **non-parenteral** prostacyclin (i.e., oral prostacyclin, inhaled prostacyclin) prior to escalating to a parenteral prostacyclin therapy?

Your best estimate will do.

[RANGE: 0-100]

1. |_|_| % of patients initiated on a non-parenteral prostacyclin prior to escalating to a parenteral prostacyclin therapy

ALL QUALIFIED RESPONDENTS (S100r1-3)
Q425 On average, how much time passes between when a patient is initiated on another type of prostacyclin (i.e., oral prostacyclin, IP receptor agonist, inhaled prostacyclin) and when they are initiated on a parenteral prostacyclin therapy?

Your best estimate will do.

[IF NUMERIC ENTRY IN ONE BOX, DO NOT FORCE THE OTHER]

[RANGE 0-50]
|_|_| years

[RANGE 0-12]
|_|_| months

99. I do not escalate patients on other types of prostacyclins to parenteral prostacyclin therapy [EXCLUSIVE]

ALL QUALIFIED RESPONDENTS (S100r1-3)
Q405 Now, we would like to focus specifically on your **intermediate risk PAH (WHO Group 1)** patients again.

Considering the **intermediate risk PAH (WHO Group 1)** patients that you currently manage, how many are currently being treated with each of the following regimens?

Your total must sum to 100%.

[RANGE: 0-100 FOR EACH; TOTAL MUST SUM TO 100; SHOW CONSTANT SUM]

	**% of intermediate risk PAH (WHO Group 1) patients on therapy**
1. One PAH-specific drug only (monotherapy)	|_|_|_| %
2. Two PAH-specific drugs (dual combination)	|_|_|_| %
3. Three or more PAH-specific drugs (triple or greater combination)	|_|_|_| %
4. Not on PAH-specific drug therapy	|_|_|_| %

ALL QUALIFIED RESPONDENTS (S100r1-3)
Q410 What proportion of your **intermediate risk** patients are currently receiving each of the following prostacyclin therapies?

Your best estimate will do. Your responses must sum to 100%.

[RANGE: 0-100 FOR EACH]

	**% of intermediate risk patients**
1. Oral treprostinil	|_|_|_| %

2. Oral selexipag |_|_|_| %
3. Inhaled treprostinil |_|_|_| %
4. Inhaled iloprost |_|_|_| %
5. Subcutaneous treprostinil |_|_|_| %
6. Intravenous treprostinil |_|_|_| %
7. Intravenous epoprostenol |_|_|_| %
8. Intermediate risk patients <u>not</u> receiving a prostacyclin drug therapy |_|_|_| %

ALL QUALIFIED RESPONDENTS (S100r1-3)
Q411 Please take a moment to review the information below regarding risk assessments using the COMPERA 2.0 methodology. In the next couple of questions, we will ask for your perceptions regarding intermediate-low versus intermediate-patients.

[INSERT PICTURE OF COMPERA 2.0 SLIDE] [HOLD ON SCREEN FOR 15 SECONDS]

Please click "Continue" when you are ready to proceed.

ALL QUALIFIED RESPONDENTS (S100r1-3)
Q412A How likely are you, personally, to initiate or recommend the following treatment regimens for **<u>intermediate-low</u> risk** patients, either as a monotherapy or in combination with other therapies?

Please click <u>here</u> for more information on intermediate-low risk classifications per the COMPERA 2.0 risk assessment methodology.

[COLUMNS]

Very unlikely	Unlikely	Neutral	Likely	Very likely
1	2	3	4	5

[ROWS]
1. PDE5/sGC
2. ERA
3. Oral prostanoid
4. Inhaled prostanoid
5. Subcutaneous prostanoid
6. Intravenous prostanoid

ALL QUALIFIED RESPONDENTS (S100r1-3)
Q412B How likely are you, personally, to initiate or recommend the following treatment regimens for **intermediate-high risk** patients, either as a monotherapy or in combination with other therapies?

Please click here for more information on intermediate-high risk classifications per the COMPERA 2.0 risk assessment methodology.

[COLUMNS]

Very unlikely	Unlikely	Neutral	Likely	Very likely
1	2	3	4	5

[ROWS]

1. PDE5/sGC
2. ERA
3. Oral prostanoid
4. Inhaled prostanoid
5. Subcutaneous prostanoid
6. Intravenous prostanoid

ALL QUALIFIED RESPONDENTS (S100r1-3)
Q440A In general, which of the following factors influence you to escalate **intermediate-low** risk PAH (WHO Group 1) patients to parenteral prostacyclin therapy?

Please click here for more information on intermediate-low risk classification per the COMPERA 2.0 risk assessment methodology.

Please select all that apply.

[RANDOMIZE, MULTI-SELECT, GROUP R2-7 IN ORDER]

1. Patient is not improving on their current therapy
2. Patient's clinical parameters are unchanged on current therapy at 3-month follow-up visit
3. Patient's clinical parameters are unchanged on current therapy at 6-month follow-up visit
4. Patient is declining on their current therapy at 3-month follow-up visit
5. Patient is declining on their current therapy at 6-month follow-up visit
6. Patient is improving on their current therapy, but has not yet met low-risk at 3-month follow-up visit
7. Patient is improving on their current therapy, but has not yet met low-risk at 6-month follow-up visit
8. Patient has good insurance coverage / can afford it
9. Patient has right heart size/structure/function parameters consistent with higher risk
10. Patient has right heart hemodynamics consistent with higher risk
11. Patient race/ethnicity

12. Patient age
13. Patient gender
14. PAH etiology
15. Patient has been on oral/inhaled prostacyclin-class therapy
16. Other, please specify: [MANDATORY TEXT BOX, ANCHOR]

ALL QUALIFIED RESPONDENTS (S100r1-3)
Q440B In general, which of the following factors influence you to escalate **intermediate-high** risk PAH (WHO Group 1) patients to parenteral prostacyclin therapy?

Please click here for more information on intermediate-high risk classification per the COMPERA 2.0 risk assessment methodology.

Please select all that apply.

[MULTI-SELECT, SHOW IN SAME ORDER AS Q440A]

1. Patient is not improving on their current therapy
2. Patient's clinical parameters are unchanged on current therapy at 3-month follow-up visit
3. Patient's clinical parameters are unchanged on current therapy at 6-month follow-up visit
4. Patient is declining on their current therapy at 3-month follow-up visit
5. Patient is declining on their current therapy at 6-month follow-up visit
6. Patient is improving on their current therapy, but has not yet met low-risk at 3-month follow-up visit
7. Patient is improving on their current therapy, but has not yet met low-risk at 6-month follow-up visit
8. Patient has good insurance coverage / can afford it
9. Patient has right heart size/structure/function parameters consistent with higher risk
10. Patient has right heart hemodynamics consistent with higher risk
11. Patient race/ethnicity
12. Patient age
13. Patient gender
14. PAH etiology
15. Patient has been on oral/inhaled prostacyclin-class therapy
16. Other, please specify: [MANDATORY TEXT BOX, ANCHOR]

ALL QUALIFIED RESPONDENTS (S100r1-3)

Q445A Which of the following factors influence you to <u>not</u> escalate **intermediate-low** risk PAH (WHO Group 1) patients to parenteral prostacyclin therapy?

Please click <u>here</u> for more information on intermediate-low risk classification per the COMPERA 2.0 risk assessment methodology.

Please select all that apply.

[RANDOMIZE, MULTI-SELECT]

1. Patients refuse treatment
2. End of life situation
3. Poor patient compliance with treatment
4. Tolerability / side effect concerns
5. Reserve use as a last resort
6. Preference to follow sequential treatment
7. Hospital/clinical guidelines or protocols
8. Limited experience of using upfront combination therapy
9. Limited clinical evidence supporting the use of upfront combination therapy
10. Patients do not need it until reaching a more severe functional class
11. Patients do not need it until reaching a high risk status
12. The patient is a better candidate for a titratable oral prostacyclin therapy
13. Cost / coverage concerns
14. Do not have time to educate patients on use
15. Contraindications
16. Patient race/ethnicity
17. Patient age
18. Patient is improving on current therapy
19. Other, please specify: [MANDATORY TEXT BOX, ANCHOR]

ALL QUALIFIED RESPONDENTS (S100r1-3)
Q445B Which of the following factors influence you to <u>not</u> escalate **intermediate-high** risk PAH (WHO Group 1) patients to parenteral prostacyclin therapy?

Please click <u>here</u> for more information on intermediate-high risk classification per the COMPERA 2.0 risk assessment methodology.

Please select all that apply.

[MULTI-SELECT, SHOW IN SAME ORDER AS Q445B]

1. Patients refuse treatment
2. End of life situation
3. Poor patient compliance with treatment
4. Tolerability / side effect concerns
5. Reserve use as a last resort
6. Preference to follow sequential treatment
7. Hospital/clinical guidelines or protocols
8. Limited experience of using upfront combination therapy
9. Limited clinical evidence supporting the use of upfront combination therapy

10. Patients do not need it until reaching a more severe functional class
11. Patients do not need it until reaching a high risk status
12. The patient is a better candidate for a titratable oral prostacyclin therapy
13. Cost / coverage concerns
14. Do not have time to educate patients on use
15. Contraindications
16. Patient race/ethnicity
17. Patient age
18. Patient is improving on current therapy
19. Other, please specify: [MANDATORY TEXT BOX, ANCHOR]

ALL QUALIFIED RESPONDENTS (S100r1-3)
Q455A How do you expect your own initiations of parenteral prostacyclins to change, if at all, over the next 12 months?

1. Significantly decrease
2. Somewhat decrease
3. Stay the same
4. Somewhat increase
5. Significantly increase

ALL QUALIFIED REPSONDENTS (S100r1-3)
Q455B Why do you expect initiations of parenteral prostacyclins to [INSERT Q455A RESPONSE IN ITALICS] over the next 12 months?

Please be as specific as possible.

[MANDATORY TEXT BOX]

SECTION 500: TREATMENT ATTITUDES – PARENTERAL THERAPY

ALL QUALIFIED RESPONDENTS (S100r1-3)
Q500 Now, we are going to be focusing on PAH parenteral prostacyclin therapy in general.

How important are each of the following factors when deciding on a specific parenteral prostacyclin therapy regimen for a patient with PAH?

Please use a scale where a "0" means "Not at all important" and a "10" means "Extremely important."

Not at all important										Extremely important
0	1	2	3	4	5	6	7	8	9	10

[RANDOMIZE]

1. Improves patient quality of life
2. Improves symptom relief
3. Allows for earlier treatment

4. Improves survival rate
5. Minimizes cost to patient
6. Minimizes tolerability issues
7. Provides dosing flexibility
8. Reduces risk status
9. Proven reliability of drug supply
10. Proven reliability of device supply
11. Improves right heart size/structure/function
12. Improves right heart hemodynamics

ALL QUALIFIED RESPONDENTS (S100r1-3)
Q505 Below is the same list you just reviewed.

Still thinking of parenteral prostacyclin therapy in general, how well do today's parenteral prostacyclin therapies meet your needs on the following factors?

Please use a scale where a "0" means "Do not at all meet my needs" and a "10" means "Completely meet my needs."

Do not at all meet my needs										Completely meet my needs
0	1	2	3	4	5	6	7	8	9	10

[SHOW IN SAME ORDER AS Q500]

1. Improves patient quality of life
2. Improves symptom relief
3. Allows for earlier treatment
4. Improves survival rate
5. Minimizes cost to patient
6. Minimizes tolerability issues
7. Provides dosing flexibility
8. Reduces risk status
9. Proven reliability of drug supply
10. Proven reliability of device supply
11. Improves right heart size/structure/function
12. Improves right heart hemodynamics

ALL QUALIFIED RESPONDENTS (S100r1-3)
Q520A We understand that there a multitude of factors considered when deciding if a patient is a candidate for parenteral prostacyclin therapy. We'd like to understand which factors are among the most influential when you are making that decision.

In your opinion, to what extent would each of the following clinical characteristics influence you to initiate a patient on parenteral prostacyclin therapy.

Please use a scale where a "1" means "No influence at all" and a "5" means "Great deal of influence."

[RANDOMIZE, GROUP R1-4 IN ORDER, GROUP R8-12 IN ORDER]

No influence at all				Great deal of influence
1	2	3	4	5

1. Patient is Functional Class I
2. Patient is Functional Class II
3. Patient is Functional Class III
4. Patient is Functional Class IV
5. Patient has abnormal right heart size/structure/function imaging parameters
6. Patient has poor right heart hemodynamics
7. Patient has been hospitalized in the past year
8. Patient is classified as low risk of death in one year
9. Patient is classified as intermediate risk of death in one year
10. Patient is classified as intermediate-low risk of death in one year
11. Patient is classified as intermediate-high risk of death in one year
12. Patient is classified as high risk of death in one year
13. For quality control purposes, please select '2'

ALL QUALIFIED RESPONDENTS (S100r1-3)
Q520C In your opinion, to what extent would each of the following patient demographics influence you to initiate a patient on parenteral prostacyclin therapy.

Please use a scale where a "1" means "No influence at all" and a "5" means "Great deal of influence."

No influence at all				Great deal of influence
1	2	3	4	5

1. Patient is younger in age (<45)
2. Patient is middle aged (45-65)
3. Patient is older in age (>65)
4. Patient is well-educated
5. Patient has commercial insurance coverage
6. Patient has Medicare/Medicaid
7. Patient has no out-of-pocket cost concerns

ALL QUALIFIED RESPONDENTS (S100r1-3)
Q520D In your opinion, to what extent would each of the following patient characteristics influence you to initiate a patient on parenteral prostacyclin therapy.

CONFIDENTIAL

Please use a scale where a "1" means "No influence at all" and a "5" means "Great deal of influence."
[RANDOMIZE]

No influence at all				Great deal of influence
1	2	3	4	5

1. Patient has cognitive impairment
2. Lack of inspiratory capacity/ability to take oral medications
3. Life expectancy
4. History of drug use
5. Patient is able to work and/or be active outside of their home
6. Patient resides close to the office/clinic for visits
7. Patient has transportation to the office/clinic for visits
8. Patient requested it
9. Patient is reliable
10. Patient has good support structure
11. Patient has dexterity issues (pump handling, drug mixing)
12. Patient is worried about site pain
13. Patient is motivated to improve physical health
14. Patient has PAH associated with connective tissue disease

ALL QUALIFIED RESPONDENTS (S100r1-3)
Q530 To what extent do you agree with the following statements?

Please use a scale where a "1" means "Strongly disagree" and a "5" means "Strongly agree."

[COLUMNS]

Strongly Disagree	Disagree	Neither agree nor disagree	Agree	Strongly agree
1	2	3	4	5

[ROWS, RANDOMIZE, CAROUSEL]

1. I aim to treat <u>all</u> appropriate PAH patients with parenteral prostacyclin therapy as soon as possible
2. I aim to treat <u>all</u> appropriate PAH patients with prostacyclin therapy (any route) as soon as possible
3. I aim to treat appropriate <u>intermediate risk</u> patients with parenteral prostacyclin therapy as soon as possible
4. I aim to treat <u>all</u> appropriate <u>intermediate risk</u> patients with prostacyclin therapy (any route) as soon as possible
5. I aim to treat appropriate <u>intermediate-low risk</u> patients with parenteral prostacyclin therapy as soon as possible
6. I aim to treat appropriate <u>intermediate-high risk</u> patients with parenteral prostacyclin therapy as soon as possible
7. If an intermediate-high risk patient is not appropriate for parenteral prostacyclin therapy, I prefer to treat them with oral prostacyclin.

8. I aim to treat appropriate <u>high risk</u> patients with parenteral prostacyclin therapy as soon as possible
9. PAH patients experience worse outcomes when parenteral prostacyclin therapy is delayed
10. I want to see continued improvement even for my PAH patients at lower risk levels
11. I tend to escalate PAH therapy as soon as possible
12. When treating PAH patients, I aim for improvement instead of stability
13. I prefer to initiate patients on an oral or inhaled prostacyclin before escalating to a parenteral prostacyclin therapy
14. Parenteral prostacyclin therapy is the most potent PAH-specific drug therapy
15. I have limited experience prescribing parenteral prostacyclin therapy
16. Parenteral prostacyclin therapy should be reserved for patients with very advanced disease progression
17. I initiate parenteral prostacyclin therapy if a patient has not reached low-risk status after 3 months on their current treatment regimen
18. I initiate parenteral prostacyclin therapy if a patient has not reached low-risk status after 6 months on their current treatment regimen

ALL QUALIFIED RESPONDENTS (S100r1-3)
QC3 For quality control purposes, please select "somewhat agree" from the list of options below.

1. Completely disagree
2. Somewhat disagree
3. Neither agree nor disagree
4. Somewhat agree
5. Completely disagree

SECTION 100: DEMOGRAPHICS

ALL QUALIFIED RESPONDENTS (S100r1-3)
Q100 As we conclude this portion of the survey, we have one final series of questions which are used for classification purposes only.

How do you describe yourself?

1. Male
2. Female
3. Non-binary

ALL QUALIFIED RESPONDENTS (S100r1-3)
Q105 In what month and year were you born?

Please enter as a four-digit number, e.g., 1963.

Month: |_|_| Year:|_|_|_|_|

[RANGE 1-12] [RANGE: 1900-2015]

ALL QUALIFIED RESPONDENTS
Q105B HIDDEN COMPUTE FOR AGE BASED ON Q105

ALL QUALIFIED RESPONDENTS (S100r1-3)
Q115 Do you currently hold an academic appointment, e.g., clinical professor, research associate, program director?

1. Yes
2. No

ALL QUALIFIED RESPONDENTS (S100r1-3)
Q120 How would you characterize the hospital with which you are primarily affiliated?

1. Major medical center, university/research hospital, or tertiary referral center
2. Community teaching hospital
3. Community non-teaching hospital
4. None of the above / my practice is not affiliated with a hospital

ALL QUALIFIED RESPONDENTS (S100r1-3)
Q125 Which description best categorizes your practice setting?

1. Urban
2. Suburban
3. Rural

SECTION P: CHART AUDIT FORM

Chart Sample Max Quota: 250

ALL RESPONDENTS
P1005 We appreciate your willingness to participate in the Pulmonary Arterial Hypertension Patient Chart Audit research!

P1010 The following is a list of information that you will be asked to enter for each of your patients. If you need time to pull the charts/information, you may suspend the survey and continue once you have the available information. We ask that you complete the chart forms as soon as possible. As a reminder, you may also have another staff member complete the chart audit form. **We will not ask for patient names, nor identify you or your patients in any way**.

- Patient characteristics, including demographics (e.g., month/year of birth, gender, comorbidities)
- PAH etiology, date of diagnosis
- Clinical parameters and assessment measurements, including, but not limited to:
    - Risk status
    - WHO/NYHA functional class
    - 6-minute walking distance (6MWD)
    - NT-proBNP/BNP plasma levels
    - Echocardiography
    - Hemodynamics (mPAP, PVR, etc.)
    - eGFR<60 mL/min (eGFR <60 mL/min/1.73m2 or defined by clinical judgement if eGFR is not available)
- PAH treatment history, date started, dosage, clinical characteristics at treatment initiation
- Vital status

ALL QUALIFIED RESPONDENTS
P1A Please take a moment again to review the COMPERA 2.0 risk assessment methodology.

Please click "Continue."

ALL QUALIFIED RESPONDENTS
P1 For the purposes of this research, we are specifically interested in patients classified as **intermediate risk** per COMPERA 2.0 who were started on a **parenteral prostacyclin** at some point thereafter with at least one comprehensive follow-up assessment while on parenteral prostacyclin therapy at which BNP/NT-proBNP, WHO FC, and 6MWD was assessed.

We are looking for patients that meet the following criteria:

- Classified as **intermediate risk** at any point between the years **2016-2020**

  - Intermediate risk status will be confirmed upon patient chart data entry.
- Initiated a parenteral prostacyclin at any time point thereafter the intermediate risk assessment
- Did not receive an experimental therapy at any point after the intermediate risk assessment
- Received a comprehensive **follow-up visit** while on parenteral prostacyclin at which clinical parameters were re-assessed **6 to 12 months after the parenteral prostacyclin was initiated**
- The following parameters must be captured at patient visits:
  - Functional Class
  - 6-minute walking distance
  - NT-proBNP or BNP plasma levels

When you are ready, please pull **2-10 patients charts** meeting the criteria detailed above. A minimum of two charts must be completed to receive compensation.

You may suspend the survey and come back to complete the chart audit when you have the information available. You will receive an honorarium for each patient chart you complete. After completing the required charts, you will be given the option to complete additional chart information.

Please click *here* if you would like to review the COMPERA 2.0 methodology again.

Please click "continue" when you are ready to proceed.

ALL QUALIFIED RESPONDENTS

P1A Should details of adverse event(s) related to our client's product(s) be mentioned during this research, are you willing to be contacted by the manufacturing company to provide additional information?

Please note: if you do not wish to be contacted, you are still able to qualify for this research.

1. Yes
2. No

[SHOW P2A–P2D ON SAME SCREEN, SHOW FOLLOWING MESSAGE AT TOP OF SCREEN]

This is chart [REF CURRENT CHART] of [10].

Before we begin, we would like to capture some baseline parameters to verify intermediate risk status according to COMPERA 2.0.

ALL QUALIFIED RESPONDENTS
P2A When was this patient first classified as intermediate risk between the years 2016-2020?

Month: |_|_| [RANGE 1-12]

Year:|_|_|_|_| [RANGE: 2016-2020]

ALL QUALIFIED RESPONDENTS
P2B At the time of this patient's intermediate risk classification, what was their WHO/NYHA Functional Class?

1. Functional Class I
2. Functional Class II
3. Functional Class III
4. Functional Class IV

ALL QUALIFIED RESPONDENTS
P2C At the time of this patient's intermediate risk classification, what was their 6-minute walking distance?

[RANGE 0-1000]

1. 6-minute walking distance: |_|_|_| meters

ALL QUALIFIED RESPONDENTS
P2D At the time of this patient's intermediate risk classification, what was their BNP or NT-proBNP levels measurement?

[RANGE 0-3000, ALLOW RESPONSE FOR BOTH P2Dr1 and P2Dr2, RESPONSE IN AT LEAST ONE ROW IS REQUIRED, DEFAULT TO R2 FOR CALCULATION]

1. BNP: |_|_|_| ng/l
2. NT-proBNP: |_|_|_| ng/l

ALL QUALIFIED RESPONDENTS
P3 HIDDEN QUESTION TO CONFIRM INTERMEDIATE RISK STATUS

Points Assigned	1	2	3	4
P2B	R1 or R2	-	R3	R4
P2C	>440	320-440	165-319	<165
P2D	R1 <50 or R2 <300	R1 50-199 or R2 300-649	R1 200-800 or R2 650-1100	R1 >800 or R2 >1100

[CALCULATION:
FOR EACH P2 VARIABLE, ASSIGN A POINT VALUE OF 1-4 PER THE RESPONSE,

THEN CALCULATE THE MEAN FROM THE 3 POINT VALUES ASSIGNED, ROUND THE MEAN TO THE NEAREST INTEGER]

[ASSIGN PER THE MEAN VALUE]

1. Low Risk (SCORE = 1) [**DISQUALIFY**]
2. Intermediate-low (SCORE = 2) [**QUALIFY**]
3. Intermediate-high (SCORE = 3) [**QUALIFY**]
4. High (SCORE =4) [**DISQUALIFY**]

NOT INTERMEDIATE RISK PATIENT OR PARENTERAL (P3r1,4)
P4A Thank you for your responses. Unfortunately, this patient does not classify as intermediate risk per the COMPERA 2.0 assessment, which is being used for this research.

Please proceed to the next screen to enter information for a new patient.

Please click *here* for more information on risk assessment using COMPERA 2.0.

[WIPE PREVIOUS P2-P3 DATA AND LOOP BACK TO P2A TO CONTINUE]

INTERMEDIATE RISK PATIENT (P3r2-3)
P2E Was this patient initiated on a parenteral prostacyclin therapy at some point following their intermediate risk classification?

1. Yes
2. No

NOT ON PARENTERAL (P2Er2)
P4B Thank you for your responses. Unfortunately, this patient does not qualify, as we are looking only for patients who were initiated on parenteral prostacyclin therapy following an intermediate risk classification.

Please proceed to the next screen to enter information for a new patient.

[WIPE PREVIOUS P2-P4A DATA AND LOOP BACK TO P2A TO CONTINUE]

QUALIFIED INTERMEDIATE RISK PARENTERAL PATIENT (P3r2-3 AND P2Er1)
P4C Thank you for your response. Please proceed to the next screen to continue with the chart form for this patient.

[CONTINUE]

[DISPLAY P5-P20 ON THE SAME SCREEN]
[ALLOW A ‘BACK’ BUTTON ON REMAINING CHART FORM QUESTIONS]

ALL QUALIFIED RESPONDENTS
P5 What is this patient’s month and year of birth?

Month: |_|_| Year:|_|_|_|_|
[RANGE 1-12] [RANGE: 1900-2015]

ALL QUALIFIED RESPONDENTS
P5A HIDDEN COMPUTE FOR AGE

ALL QUALIFIED RESPONDENTS
P10 What is this patient's sex?

1. Male
2. Female
3. Other

ALL QUALIFIED RESPONDENTS
P15 Which of the following best describes this patient's racial or ethnic background?

Please select all that apply.

[ALPHA SORT, MULTI-SELECT]

1. African American or Black
2. Asian or South Asian
3. Caucasian or White
4. Hispanic/Latino
5. Middle Eastern or Northern African
6. Native Hawaiian or other Pacific Islander
7. Native American or Alaska Native
8. Another race/ethnicity, please specify: [TEXT BOX] [ANCHOR]

ALL QUALIFIED RESPONDENTS
P20 With what comorbidities has this patient been diagnosed?

Please select all that apply.

[ALPHA SORT, MULTI-SELECT]

1. Substance abuse
2. Autoimmune disorders
3. HIV
4. Chronic liver disease
5. COPD
6. Depression
7. Hypertension
8. Obesity
9. Type 2 diabetes mellitus
10. Sleep apnea
11. Thyroid disease

12. Hyperlipidemia
13. Left heart failure with reduced or preserved ejection fraction
14. Coronary artery disease
15. Anemia
16. Asthma
17. Interstitial lung disease
18. Valvular disease
19. Other condition, please specify: [ANCHOR] [OPEN TEXT BOX]
20. No comorbidities [EXCLUSIVE] [ANCHOR]

ALL QUALIFIED RESPONDENTS
P25 When did you start managing this patient?

Month: |_|_| Year:|_|_|_|_|
[RANGE 1-12] [RANGE: 1970-2020]

[P25 MUST BE AFTER P5, THEIR DATE OF BIRTH]

99 Not sure [EXCLUSIVE]

ALL QUALIFIED RESPONDENTS
P25A HIDDEN QUESTION TO DETERMINE TIME MANAGED BY CURRENT HCP

[CALCULATE THE NUMBER OF MONTHS FROM P25 TO TODAY]

ALL QUALIFIED RESPONDENTS
P35 When was this patient diagnosed with PAH?

Month: |_|_| Year:|_|_|_|_|
[RANGE 1-12] [RANGE: 1900-2020]

[P35 MUST BE AFTER P5, THEIR DATE OF BIRTH]
[P35 MUST BE BEFORE OR SAME AS P2A, THEIR INDEX VISIT]

99 Not sure [EXCLUSIVE]

ALL QUALIFIED RESPONDENTS
P35B HIDDEN QUESTION TO DETERMINE TIME SINCE DIAGNOSIS

[CALCULATE THE NUMBER OF MONTHS FROM P35 TO TODAY]. IF 99 CLASSIFY AS "NOT SURE"

[DISPLAY P37 AND P38 ON SAME SCREEN]
ALL QUALIFIED RESPONDENTS
P37 Which of the following best describes this patient's PAH etiology?

[SINGLE-SELECT, ALPHA SORT]
1. Idiopathic (IPAH)
2. Heritable
3. Drug- and toxin induced

4. PAH associated with connective tissue disease (CTD-PAH)
5. PAH associated with HIV infection
6. PAH associated with portal hypertension (PoPH)
7. PAH associated with congenital heart disease
8. PAH associated with other conditions [ANCHOR]

ALL QUALIFIED RESPONDENTS

P38 What was this patient's WHO/NYHA functional class at the **time of diagnosis**?

1. Functional Class I
2. Functional Class II
3. Functional Class III
4. Functional Class IV
5. Unknown /Not recorded

[DISPLAY P42, P50, P49, P48, P52 ON THE SAME SCREEN]

[DISPLAY THE FOLLOWING AT THE TOP OF SCREEN: "*For the next set of questions, we'd like to capture various clinical parameters that were assessed around the time of this patient's* *<u>intermediate risk classification</u>. Parameters that were captured **within 30-60 days** of this patient's intermediate risk classification are appropriate.*"]

At the time of this patient's intermediate risk classification…

ALL QUALIFIED RESPONDENTS

P42 Were they…?

1. Unchanged (*<u>neither</u> improving nor declining since their previous visit*)
2. Improving (*<u>improvement</u> in disease progression since their previous visit*)
3. Declining (*<u>decline</u> in disease progression since their previous visit*)
4. Unknown / Not recorded

ALL QUALIFIED RESPONDENTS

P50 What was their <u>heart rate (BPM)</u>?

1. HR, BPM > 96
2. HR, BPM ≤ 96
3. Unknown / Not recorded within 30-60 days of intermediate risk classification

ALL QUALIFIED RESPONDENTS

P49 What was their <u>systolic blood pressure (SBP)</u>?

1. SBP ≥ 110 mmHg
2. SBP < 110 mmHg
3. Unknown / Not recorded within 30-60 days of intermediate risk classification

ALL QUALIFIED RESPONDENTS
P48 Did they have an eGFR measurement of <60mL/min/1.73m^2 or renal insufficiency?

1. Yes
2. No
3. Unknown / Not recorded within 30-60 days of intermediate risk classification

ALL QUALIFIED RESPONDENTS
P52 What was their measure of diffusing capacity for carbon monoxide (DLCO)?

1. % predicted DLCO <40
2. % predicted DLCO ≥40
3. Unknown /Not recorded

ALL QUALIFIED RESPONDENTS
P67 You mentioned that this patient was classified as intermediate risk in [ENTER MONTH/YEAR FROM P2A].

When was this patient's most recent **right heart catheterization** in proximity to their intermediate risk classification?

Month: |_|_| Year:|_|_|_|_|
[RANGE 1-12] [RANGE: 1900-2023]

[P67 MUST BE SAME OR AFTER THEIR DIAGNOSIS P35. IF P35r99 SELECTED, P67 MUST BE AFTER DATE OF BIRTH (P5)]

99. This patient has never received a right heart catheterization [EXCLUSIVE]

[SHOW P46-P59 ON SAME SCREEN]
After receiving a right heart catheterization in [ENTER P67 DATE]...

RHC RECEIVED (P67/NE 99)
P46 What was their pulmonary vascular resistance (PVR)?

[R1: ALLOW DECIMAL ENTRY, RANGE 0-20]
[R2: RANGE 0-999]
[ALLOW RESPONSE IN R1 OR R2, DO NOT REQUIRE BOTH]

1. PVR: |_|_|_| Wood units (WU)
2. PVR: |_|_|_| dynes/sec/cm^{-5}

99. Unknown / Not recorded

RHC RECEIVED (P67/NE 99)
P47 What was their measure of mean pulmonary artery pressure (mPAP)?

[RANGE 0-100]

1. mPAP: |_|_|_| mmHg
99. Unknown / Not recorded

RHC RECEIVED (P67/NE 99)
P53 What was their measure of mean right atrial pressure (mRAP)?

1. >20 mmHg
2. ≤20 mmHg
3. Unknown /Not recorded

RHC RECEIVED (P67/NE 99)
P57 What was their cardiac index?

[RANGE: 0-50, ALLOW DECIMAL VALUE]

1. |_|_|_| L/min/m^2
2. Unknown /Not recorded

RHC RECEIVED (P67/NE 99)
P58 What was their cardiac output?

[RANGE: 0-50, ALLOW RESPONSE IN ONLY ONE ROW, ALLOW DECIMAL VALUE]

1. Thermodilution measurement: |_|_|_| L/min
2. Fick measurement: |_|_|_| L/min
3. Unknown / Not recorded

RHC RECEIVED (P67/NE 99)
P59 What was their measure of stroke volume (SV)?

[RANGE: 0-999]

1. |_|_|_| mL
2. Unknown / Not recorded

ALL QUALIFIED RESPONDENTS
P68 You mentioned that this patient was classified as intermediate risk in [ENTER MONTH/YEAR FROM P2A].

Now, when was this patient's most recent **echocardiogram** in proximity to their intermediate risk classification?

Month: |_|_| [RANGE 1-12] Year:|_|_|_|_| [RANGE: 1900-2023]

[P68 MUST BE SAME OR AFTER THEIR DIAGNOSIS P35. IF P35r99 SELECTED, P67 MUST BE AFTER DATE OF BIRTH (P5)]]

99. This patient has never received an echocardiogram [EXCLUSIVE]

[SHOW P51-P70 ON SAME SCREEN]
After receiving an echocardiogram in [ENTER P68 DATE]…

ECHO RECEIVED (P68/NE 99)
P51 Did the echocardiogram show pericardial effusion?

1. None
2. Minimal
3. Moderate or large
4. Unknown / Not recorded

ECHO RECEIVED (P68/NE 99)
P61 How would you define their RV function?

1. Normal
2. Mildly reduced
3. Moderately reduced
4. Severely reduced
5. Unknown / Not recorded

ECHO RECEIVED (P68/NE 99)
P71 How would you define their RA size?

1. Normal
2. Mildly enlarged
3. Moderately enlarged
4. Severely enlarged
5. Unknown / Not recorded

ECHO RECEIVED (P68/NE 99)
P72 How would you define their RV size?

1. Normal
2. Mildly enlarged
3. Moderately enlarged
4. Severely enlarged
5. Unknown / Not recorded

ECHO RECEIVED (P68/NE 99)
P62 What was their measure of tricuspid annular plane systolic excursion (TAPSE)?

[RANGE: 0-999, ALLOW DECIMAL RESPONSES]

1. |_|_|_| mm
2. Unknown / Not recorded

ECHO RECEIVED (P68/NE 99)
P69 What was their measure of systolic pulmonary arterial pressure (sPAP)?

[RANGE: 0-999, ALLOW DECIMAL RESPONSES]

1. |_|_|_| mmHg
2. Unknown / Not recorded

ECHO RECEIVED (P68/NE 99)
P66 What was their tricuspid regurgitation (TR) severity?

1. None
2. Mild
3. Moderate
4. Severe
5. Unknown / Not recorded

ECHO RECEIVED (P68/NE 99)
P70 Did the echocardiogram show systolic interventricular septal flattening?

1. Yes
2. No
3. Unknown / Not recorded

ALL QUALIFIED RESPONDENTS
P95 Now, when did this patient start a parenteral prostacyclin therapy following their intermediate risk classification?

Month: |_|_| Year:|_|_|_|_|

[RANGE 1-12] [RANGE: 2016-2023]

[P95 MUST BE AFTER OR SAME AS P2A, THEIR INDEX VISIT]

Not sure [EXCLUSIVE] [HIDE]

[DISPLAY P85-P90 ON THE SAME SCREEN]
ALL QUALIFIED RESPONDENTS
P85 Which of the following parenteral prostacyclin therapy was this patient initiated on following their intermediate risk classification?

[SINGLE-SELECT, RANDOMIZE, GROUP R1-2]
1. Subcutaneous treprostinil
2. Intravenous treprostinil
3. Intravenous epoprostenol

ALL QUALIFIED RESPONDENTS
P86 Which of the following therapies was this patient taking in combination, if any, in addition to their parenteral prostacyclin therapy?

Please select all that apply.
[MULTI-SELECT, KEEP GROUPS IN ORDER - RANDOMIZE R1-3, RANDOMIZE R4-5, RANDOMIZE R6-7]
1. Oral PDE5 inhibitors (tadalafil, sildenafil)
2. Oral ERAs (bosentan, ambrisentan, macitentan)
3. sGC stimulators (riociguat)
4. Other, please specify [TEXT BOX] [ANCHOR]
5. This patient was not taking any additional therapies in combination with their parenteral prostacyclin therapy [EXCLUSIVE] [ANCHOR]

ALL QUALIFIED RESPONDENTS
P86B Did this patient transition to their parenteral prostacyclin from another non-parenteral prostacyclin? If so, which prostacyclin did they transition from?

[ALPHA SORT]
1. Oral treprostinil
2. Oral selexipag
3. Inhaled treprostinil
4. Inhaled iloprost
5. Other, please specify: [TEXT BOX] [ANCHOR]
6. No, this patient did <u>not</u> transition from another prostacyclin [ANCHOR]

ALL QUALIFIED RESPONDENTS
P90 What are the main reasons this patient was initiated on this parenteral prostacyclin therapy? Please be as detailed as possible.

[MANDATORY TEXT BOX]

[DISPLAY P96A-P96C ON THE SAME SCREEN]
ALL QUALIFIED RESPONDENTS
P96A What was this patient's most recent WHO/NYHA functional class at the **time this <u>parenteral</u> prostacyclin therapy was initiated**?

If this assessment was not conducted exactly at the time of parenteral prostacyclin initiation, please refer to the assessment conducted in closest proximity to this time.

1. Functional Class I
2. Functional Class II
3. Functional Class III
4. Functional Class IV
5. Unknown /Not recorded [HIDE]

ALL QUALIFIED RESPONDENTS
P96B What was this patient's most recent 6-minute walking distance at the **time this <u>parenteral</u> prostacyclin therapy was initiated**?

If this assessment was not conducted exactly at the time of parenteral prostacyclin initiation, please refer to the assessment conducted in closest proximity to this time.

[RANGE: 0-1000]
1. 6-minute walking distance: |_|_|_| meters
2. Unknown /Not recorded [EXCLUSIVE] [HIDE]

ALL QUALIFIED RESPONDENTS
P96C What was this patient's most recent BNP or NT-proBNP levels measurement at the **time this <u>parenteral prostacyclin</u> therapy was initiated**?

If this assessment was not conducted exactly at the time of parenteral prostacyclin initiation, please refer to the assessment conducted in closest proximity to this time.

[RANGE 0-3000, RESPONSE IN AT LEAST ONE ROW IS REQUIRED, RESPONENT DOES NOT HAVE TO RESPOND TO BOTH R1 AND R2]

1. BNP: |_|_|_| ng/l
2. NT-proBNP: |_|_|_| ng/l
3. Unknown /Not recorded [EXCLUSIVE] [HIDE]

ALL QUALIFIED RESPONDENTS
P98A What was the <u>initial starting</u> dose for this patient's **<u>parenteral prostacyclin therapy</u> at the time it was initiated?**

Please provide your best estimate.

[DISPLAY ROW BASED ON P85 SELECTION, ALLOW DECIMAL RESPONSES]

1. Subcutaneous treprostinil |_|_|_|_| ng/kg/min
2. Intravenous treprostinil |_|_|_|_| ng/kg/min
3. Intravenous epoprostenol |_|_|_|_| ng/kg/min
4. Unknown /Not recorded [EXCLUSIVE]

ALL QUALIFIED RESPONDENTS
P100 *For the next set of questions, we'd like to capture information about this patient's* ***comprehensive follow-up visit <u>6 to 12 months</u>*** *after starting parenteral prostacyclin therapy.*

We are looking for a full follow-up visit at which comprehensive clinical assessments (WHO FC, 6MWD, NT-proBNP/BNP, imaging, etc.) were performed, as opposed to short-term check-ins (e.g., monitoring tolerability, compliance, etc.).

ALL QUALIFIED RESPONDENTS
P140 Now, when was this patient's comprehensive follow-up visit 6 to 12 months after being initiated on the parenteral prostacyclin?

By "comprehensive follow-up visit," we mean a visit at which comprehensive clinical assessments (WHO FC, 6MWD, NT-proBNP/BNP, imaging, etc.) were performed, as opposed to a short-term check-in.

Month: |_|_| Year:|_|_|_|_|
[RANGE 1-12] [RANGE: 2016-2023]

[P140 MUST BE BETWEEN 6 – 12 MONTHS AFTER P95, THEIR PARENTERAL THERAPY INITIATION]

99 Not sure [EXCLUSIVE] [HIDE]

[DISPLAY P141-P145 ON THE SAME SCREEN]
[DISPLAY THE FOLLOWING AT THE TOP OF SCREEN:

At the time of this patient's follow-up visit…

ALL QUALIFIED RESPONDENTS
P141 What was their WHO/NYHA Functional Class?

1. Functional Class I
2. Functional Class II
3. Functional Class III
4. Functional Class IV

ALL QUALIFIED RESPONDENTS
P144 What was their 6-minute walking distance?

[RANGE 0-1000]

1. 6-minute walking distance: |_|_|_| meters

ALL QUALIFIED RESPONDENTS
P145 What was their BNP or NT-proBNP plasma levels measurement?

[RANGE 0-3000, RESPONSE IN AT LEAST ONE ROW IS REQUIRED, RESPONENT DOES NOT HAVE TO RESPOND TO BOTH R1 AND R2]

1. BNP: |_|_|_| pg/mL
2. NT-proBNP: |_|_|_| pg/mL

[DISPLAY P142-P152 ON THE SAME SCREEN]
[DISPLAY THE FOLLOWING AT THE TOP OF SCREEN: "*Again, for this next set of questions, we'd like to capture various clinical parameters that were assessed at the time of this patient's follow-up visit. Parameters that were captured within 30-60 days of the follow-up visit are appropriate.*"]

At the time of this patient's follow-up visit…

ALL QUALIFIED RESPONDENTS
P142 Were they…?

1. Unchanged (*neither improving nor declining since their previous visit*)
2. Improving (*improvement in disease progression since their previous visit*)
3. Declining (*decline in disease progression since their previous visit*)
4. Unknown / Not recorded

ALL QUALIFIED RESPONDENTS
P150 What was their heart rate (BPM)?

1. HR, BPM > 96
2. HR, BPM ≤ 96
3. Unknown / Not recorded within 30-60 days of follow-up visit

ALL QUALIFIED RESPONDENTS
P149 What was their systolic blood pressure (SBP)?

1. SBP ≥ 110 mmHg
2. SBP < 110 mmHg

Unknown / Not recorded within 30-60 days of follow-up visit

ALL QUALIFIED RESPONDENTS
P148 Did they have an eGFR measurement of <60mL/min/1.73m^2 or renal insufficiency?

1. Yes
2. No
3. Unknown / Not recorded within 30-60 days of follow-up visit

ALL QUALIFIED RESPONDENTS
P152 What was their measure of diffusing capacity for carbon monoxide (DLCO)?

1. % predicted DLCO <40
2. % predicted DLCO ≥40
3. Unknown /Not recorded

ALL QUALIFIED RESPONDENTS
P167 You mentioned that this patient received a right heart catheterization in [ENTER MONTH/YEAR FROM P67], in proximity to their intermediate risk classification.

Was a **right heart catheterization** repeated, in proximity to their comprehensive follow-up visit? If so, when?

Month: |_|_| [RANGE 1-12]   Year:|_|_|_|_| [RANGE: 1900-2023]

[P167 MUST BE SAME OR AFTER PREVIOUS RHC P67]

99. This patient did not receive another right heart catheterization [EXCLUSIVE]

[SHOW P46-P59 ON SAME SCREEN]
After receiving a right heart catheterization in [ENTER P167 DATE]…

RHC RECEIVED (P67/NE 99)
P146 What was their pulmonary vascular resistance (PVR)?

[R1: ALLOW DECIMAL ENTRY, RANGE 0-20]
[R2: RANGE 0-999]
[ALLOW RESPONSE IN R1 OR R2, DO NOT REQUIRE BOTH]

1. PVR: |_|_|_| Wood units (WU)
2. PVR: |_|_|_| dynes/sec/cm^{-5}

99. Unknown / Not recorded

RHC RECEIVED (P67/NE 99)
P147 What was their measure of mean pulmonary artery pressure (mPAP)?

[RANGE 0-100]

1. mPAP: |_|_|_| mmHg

99. Unknown / Not recorded

RHC RECEIVED (P67/NE 99)
P153 What was their measure of mean right atrial pressure (mRAP)?

1. >20 mmHg
2. ≤20 mmHg
3. Unknown /Not recorded

RHC RECEIVED (P67/NE 99)
P157 What was their cardiac index?

[RANGE: 0-50, ALLOW DECIMAL VALUE]

1. |_|_|_| L/min/m^2
2. Unknown /Not recorded

RHC RECEIVED (P67/NE 99)
P158 What was their cardiac output?

[RANGE: 0-50, ALLOW RESPONSE IN ONLY ONE ROW, ALLOW DECIMAL RESPONSE]

1. Thermodilution measurement: |_|_|_| L/min
2. Fick measurement: |_|_|_| L/min
3. Unknown / Not recorded

RHC RECEIVED (P67/NE 99)
P159 What was their measure of stroke volume (SV)?

[RANGE: 0-999]

1. |_|_|_| mL
2. Unknown / Not recorded

ALL QUALIFIED RESPONDENTS
P168 You mentioned that this patient received an echocardiogram in [ENTER MONTH/YEAR FROM P68], in proximity to their intermediate risk classification.

Was an **echocardiogram** repeated, in proximity to their comprehensive follow-up visit? If so, when?

Month: |_|_| Year:|_|_|_|_|
[RANGE 1-12] [RANGE: 1900-2023]

[P168 MUST BE SAME OR AFTER PREVIOUS ECHO P68]

99. This patient did not receive another echocardiogram [EXCLUSIVE]

[SHOW P151-P170 ON SAME SCREEN]
After receiving an echocardiogram in [ENTER P168 DATE]…

ECHO RECEIVED (P68/NE 99)
P151 Did the echocardiogram show pericardial effusion?

1. None
2. Minimal
3. Moderate or large
4. Unknown / Not recorded

ECHO RECEIVED (P68/NE 99)
P161 How would you define their RV function?

1. Normal
2. Mildly reduced
3. Moderately reduced
4. Severely reduced
5. Unknown / Not recorded

ECHO RECEIVED (P68/NE 99)
P171 How would you define their RA size?

1. Normal
2. Mildly enlarged
3. Moderately enlarged
4. Severely enlarged
5. Unknown / Not recorded

ECHO RECEIVED (P68/NE 99)
P172 How would you define their RV size?

1. Normal
2. Mildly enlarged
3. Moderately enlarged
4. Severely enlarged
5. Unknown / Not recorded

ECHO RECEIVED (P68/NE 99)
P162 What was their measure of tricuspid annular plane systolic excursion (TAPSE)?

[RANGE: 0-999, ALLOW DECIMAL RESPONSES]

1. |_|_|_| mm

2. Unknown / Not recorded

ECHO RECEIVED (P68/NE 99)
P169 What was their measure of systolic pulmonary arterial pressure (sPAP)?

[RANGE: 0-999, ALLOW DECIMAL RESPONSES]
1. |_|_|_| mmHg
2. Unknown / Not recorded

ECHO RECEIVED (P68/NE 99)
P166 What was their tricuspid regurgitation (TR) severity?

1. None
2. Mild
3. Moderate
4. Severe
5. Unknown / Not recorded

ECHO RECEIVED (P68/NE 99)
P170 Did the echocardiogram show systolic interventricular septal flattening?

1. Yes
2. No
3. Unknown / Not recorded

ALL QUALIFIED RESPONDENTS
P198 At the time of this patient's follow-up visit, what was the dose of this patient's parenteral prostacyclin therapy?

Please provide your best estimate. If you did not capture this patient's dose at the time of this follow-up visit, please provide this patient's most recent dosage in proximity to the follow-up visit.

[LIST EACH DRUG SELECTED AT P85, ALLOW DECIMAL RESPONSES]
1. Subcutaneous treprostinil |_|_|_|_| ng/kg/min
2. Intravenous treprostinil |_|_|_|_| ng/kg/min
3. Intravenous epoprostenol |_|_|_|_| ng/kg/min
4. Unknown /Not recorded [EXCLUSIVE]

[SHOW P198B AS POP-UP ON SAME SCREEN AS P198]

PROVIDES DOSE (P198/NE 4)
P198B When was this dose captured?

1. At the time of the follow-up visit [EXCLUSIVE]

2. At another time:
   Month: |_|_| Year:|_|_|_|_|
   [RANGE 1-12]   [RANGE: 2016-2023]
   [P198B MUST BE SAME OR AFTER P95]

ALL QUALIFIED RESPONDENTS
P199 Is this patient still taking their parenteral prostacyclin therapy?

1. Yes
2. No

PATIENT DISCONTINUED PARENTERAL (P199r2)
P199B When did this patient stop taking their parenteral prostacyclin therapy?

Month: |_|_| Year:|_|_|_|_|
[RANGE 1-12]   [RANGE: 2016-2023]

[DATE MUST BE AFTER OR SAME AS P140]

ALL QUALIFIED RESPONDENTS
P220 Now, we are interested in the rest of this patient's treatment history through the time of their comprehensive follow-up.

Please select from the options below which **<u>other</u>** PAH-specific drugs, besides [INSERT P85], this patient has received, along with their start and stop dates

[COLUMNS]
[CHECK THAT ALL DATES ARE AFTER OR SAME AS DIAGNOSIS DATE (P35)]
[CHECK THAT STOP DATE (C2) IS AFTER OR SAME AS START DATE (C1) FOR EACH ROW IF NON-BLANK]

1. Start date:

   Month: |_|_| Year:|_|_|_|_|
   [RANGE 1-12]   [RANGE: 1900-2023]

2. Stop date:

   Month: |_|_| Year:|_|_|_|_| |_| Patient is still taking this therapy
   [RANGE 1-12]   [RANGE: 1900-2023]

[ROWS] [DO NOT SHOW SELECTION AT P85 CORRESPONDING TO R10-12]
1. Tadalafil
2. Sildenafil
3. Bosentan
4. Ambrisentan
5. Macitentan

6. Riociguat
7. Oral treprostinil
8. Oral selexipag
9. Inhaled treprostinil
10. Subcutaneous treprostinil
11. Intravenous treprostinil
12. Intravenous epoprostenol
13. Other, please specify [TEXT BOX]

ALL QUALIFIED RESPONDENTS
P300 Is this patient alive or deceased at the time of patient chart completion?

1. Alive
2. Deceased

[SHOW P301 AND P302 ON P300 SCREEN. IF P300R2 SELECTED, DISPLAY "PLEASE PROVIDE THE FOLLOWING INFORMATION" ON TOP OF P301 AND P302]

PATIENT DECEASED (P300r2)
P301 Date of death:

Month: |_|_| [RANGE 1-12]   Year:|_|_|_|_| [RANGE: 2016-2023]

[P301 MUST BE AFTER P140, THEIR FOLLOW-UP VISIT]

99 Not sure [EXCLUSIVE]

PATIENT DECEASED (P300r2)
P302 Reason for death:

1. PAH-related
2. Non-PAH related
3. Not sure

ALL QUALIFIED RESPONDENTS
P305 Do you have another patient chart to complete?

1. Yes
2. No

[IF YES (P305r1), LOOP BACK TO P2 TO RESTART CHART AUDIT FOR UP TO 10 CHARTS.]

[CHART QUOTA = 250]
[CHART QUOTA PER RESPONDENT =10]

COMPLETED PATIENT CHARTS (COMPLETED 10 CHARTS OR P305r2)

P310 Thank you for completing this survey and patient chart exercise! In the next few months, we will be conducting **60-minute** **in-depth interviews using** **Zoom** with individuals like yourself to learn more about the use of parenteral prostacyclin therapy use in PAH patients.

Are you interested in participating? If so, we will reach back out to you at a later date with more details.

1. Yes, I am interested in participating
2. No, I am not interested in participating
