# Supplementary material for: Parenteral prostacyclin utilization in patients with pulmonary arterial hypertension in the intermediate-risk strata: a retrospective chart review and cross-sectional survey
Source: BMC Pulm Med. 2024 Nov 20;24:574. doi: 10.1186/s12890-024-03388-w (PMC11577822; doi:10.1186/s12890-024-03388-w)
Supplement: Supplementary file 2 — Supplementary Material 2 [file 12890_2024_3388_MOESM2_ESM.pptx]

## Slide 1
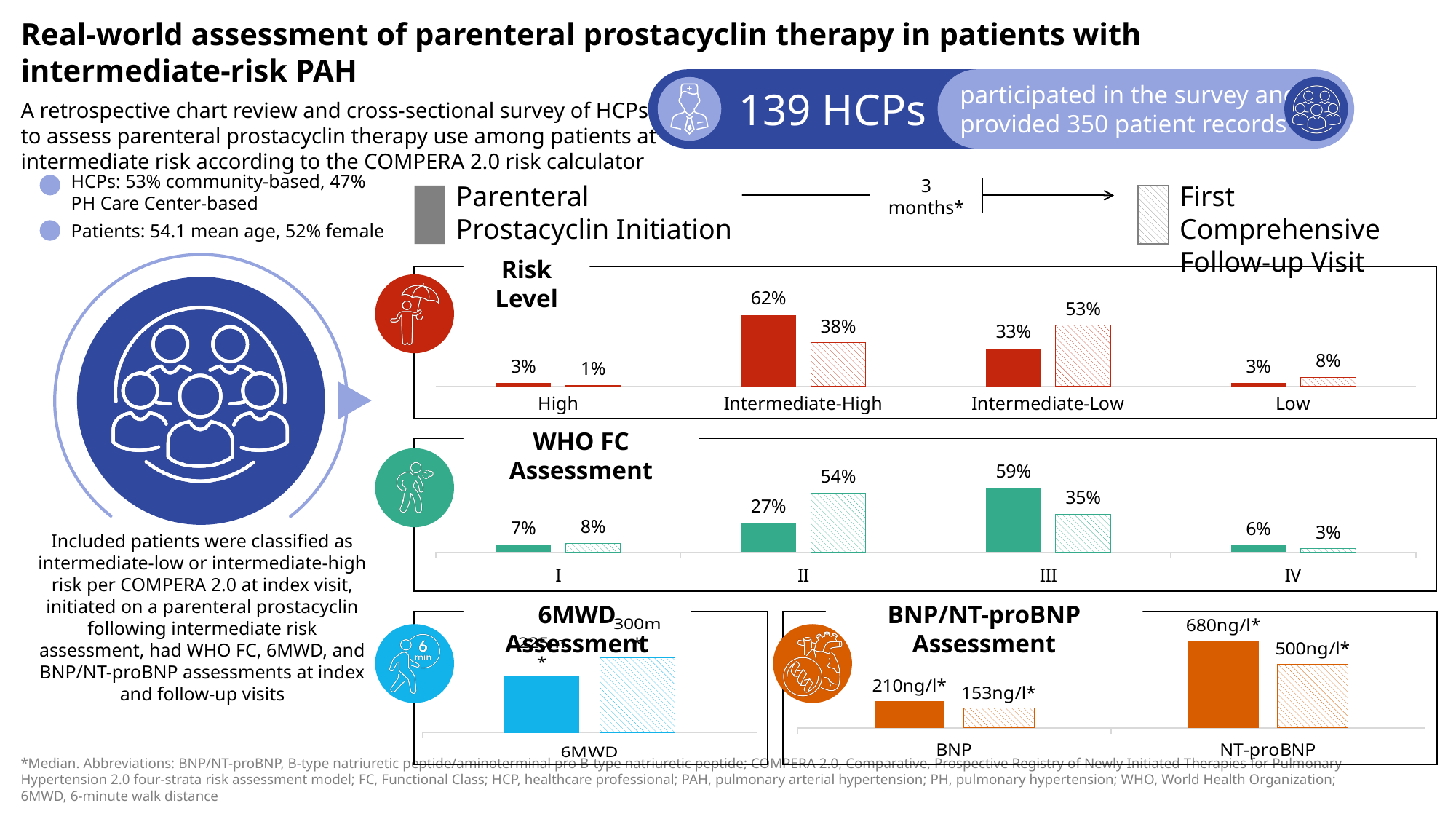

Real-world assessment of parenteral prostacyclin therapy in patients with intermediate-risk PAH
A retrospective chart review and cross-sectional survey of HCPs
to assess parenteral prostacyclin therapy use among patients at
intermediate risk according to the COMPERA 2.0 risk calculator
139 HCPs
participated in the survey and provided 350 patient records
HCPs: 53% community-based, 47% PH Care Center-based
Parenteral Prostacyclin Initiation
First Comprehensive Follow-up Visit
3 months*
Patients: 54.1 mean age, 52% female
Risk Level
### Chart
| Category | Series 1 | Series 2 |
|---|---|---|
| High | 0.03 | 0.01 |
| Intermediate-High | 0.62 | 0.38 |
| Intermediate-Low | 0.33 | 0.53 |
| Low | 0.03 | 0.08 |
WHO FC Assessment
### Chart
| Category | Series 1 | Series 2 |
|---|---|---|
| I | 0.07 | 0.08 |
| II | 0.27 | 0.54 |
| III | 0.59 | 0.35 |
| IV | 0.06 | 0.03 |
Included patients were classified as intermediate-low or intermediate-high risk per COMPERA 2.0 at index visit, initiated on a parenteral prostacyclin following intermediate risk assessment, had WHO FC, 6MWD, and BNP/NT-proBNP assessments at index and follow-up visits
6MWD Assessment
BNP/NT-proBNP Assessment
### Chart
| Category | Series 1 | Series 2 |
|---|---|---|
| 6MWD | 225.0 | 300.0 |
### Chart
| Category | Series 1 | Series 2 |
|---|---|---|
| BNP | 210.0 | 153.0 |
| NT-proBNP | 680.0 | 500.0 |
*Median. Abbreviations: BNP/NT-proBNP, B-type natriuretic peptide/aminoterminal pro B-type natriuretic peptide; COMPERA 2.0, Comparative, Prospective Registry of Newly Initiated Therapies for Pulmonary Hypertension 2.0 four-strata risk assessment model; FC, Functional Class; HCP, healthcare professional; PAH, pulmonary arterial hypertension; PH, pulmonary hypertension; WHO, World Health Organization; 6MWD, 6-minute walk distance
